# The New Unified Theory of ATP Synthesis/Hydrolysis and Muscle Contraction, Its Manifold Fundamental Consequences and Mechanistic Implications and Its Applications in Health and Disease

**DOI:** 10.3390/ijms9091784

**Published:** 2008-09-17

**Authors:** Sunil Nath

**Affiliations:** Department of Biochemical Engineering and Biotechnology, Indian Institute of Technology, Delhi, Hauz Khas, New Delhi 110016, India

**Keywords:** Bioenergetics, F_1_F_O_-ATP synthase, Myosin and kinesin, Mechanochemistry, Energy transduction, Torsional mechanism of energy transduction and ATP synthesis, Rotation-uncoiling-tilt (RUT) energy storage mechanism of muscle contraction, Quantized theory of biological molecular machines, Apoptosis, Molecular systems biology

## Abstract

Complete details of the thermodynamics and molecular mechanisms of ATP synthesis/hydrolysis and muscle contraction are offered from the standpoint of the torsional mechanism of energy transduction and ATP synthesis and the rotation-uncoiling-tilt (RUT) energy storage mechanism of muscle contraction. The manifold fundamental consequences and mechanistic implications of the unified theory for oxidative phosphorylation and muscle contraction are explained. The consistency of current mechanisms of ATP synthesis and muscle contraction with experiment is assessed, and the novel insights of the unified theory are shown to take us beyond the binding change mechanism, the chemiosmotic theory and the lever arm model. It is shown from first principles how previous theories of ATP synthesis and muscle contraction violate both the first and second laws of thermodynamics, necessitating their revision. It is concluded that the new paradigm, ten years after making its first appearance, is now perfectly poised to replace the older theories. Finally, applications of the unified theory in cell life and cell death are outlined and prospects for future research are explored. While it is impossible to cover each and every specific aspect of the above, an attempt has been made here to address all the pertinent details and what is presented should be sufficient to convince the reader of the novelty, originality, breakthrough nature and power of the unified theory, its manifold fundamental consequences and mechanistic implications, and its applications in health and disease.

## 1. Introduction

As research subjects the molecular mechanism of ATP synthesis and the molecular mechanism of muscle contraction have intrigued and captured the attention of several generations of scientists during the past ∼75 and ∼150 years, respectively, and a tremendous amount of experimental and theoretical work (that never ceases to defy this researcher’s imagination) has been done (for selected, longer, and more comprehensive reviews in this decade, see [1–5]). Yet, incredibly, this subject is still not fully understood, and several central fundamental issues remain unexplained, key aspects explored but failed to be resolved with finality, or not considered in adequate detail. Several stalwart researchers have dedicated their life to these problems [6–9] and continue to inspire younger generations of scientists. It may be possible for us to “stand on the shoulders of giants” and see beyond current paradigms, and by setting up novel research programs (along with some good fortune) to look at these difficult problems in a fresh and imaginative way that may lead to a better understanding, surmount the present obstacles facing us, and hopefully, even solve the enigmas and riddles we have been confronted with in these important fields of scientific endeavor.

One such research program on the mechanism and thermodynamics of molecular machines funded during the past ∼15 years has led to new thinking and analysis through the conception and detailed formulation of the torsional mechanism of energy transduction and ATP synthesis and the rotation-uncoiling-tilt (RUT) energy storage mechanism of muscle contraction [1, 2, 10–29]. In this paper, further subtle but crucial, fundamental details of the molecular mechanisms of ATP synthesis and muscle contraction and their consequences that had remained inaccessible are revealed from the standpoint of the torsional and RUT mechanisms and unification of the thermodynamic ATP cycle. Some current theories are shown to be in disagreement with important biochemical and biophysical (including single molecule) data. Moreover, in a very simple way, it is shown why and how previous theories violate both the first and second laws of thermodynamics and hence require revision. Such difficulties are not present in the new paradigm, which is now perfectly poised to replace the older theories. Finally, emphasis is laid on the biological applications of the unified theory in cell life and death, and how several scientific doors can be opened leading to new vistas and opportunities for future research.

## 2. Unified Theory of the ATP Cycle in ATP Synthesis/Hydrolysis and Muscle Contraction

### 2.1. Specific Details of Energy Distribution among the Elementary Steps of Binding, Bond Cleavage, and Product Release in the ATP Synthesis/Hydrolysis Catalytic Cycle

All the membrane-bound/organized biological molecular machines that we are concerned with here, such as the F_1_F_O_-ATP synthase, the P- and V-type ATPases, muscle myosin II, the cargo-carrying kinesins and unconventional myosins etc., synthesize or utilize ATP. Hence it would be valuable for a unified theory if we can understand the quantum of energy required/released in each elementary step of the ATP cycle in synthesis/hydrolysis. This by itself would be an all-consuming exercise, but it can be made tractable for the present work by selecting as an appropriate starting point the section on physical, chemical and biological implications of Ref. [10], especially column 2 on p. 2232 [10]. As emphasized there, we can have different theories depending on how we redistribute the standard state Gibbs free energy of the ATP-ADP couple among the various elementary steps of binding, bond formation/breakage, and release. As clearly mentioned there (in the context of the rotation of the central γ-subunit in F_1_-ATPase/rotation of the myosin head in muscle myosin), “whether ATP binding energy, or ATP hydrolysis, or both cause rotation in the hydrolysis mode does not present problems for the torsional or RUT mechanism.” This was because the bond cleavage step immediately followed the ATP binding step on the enzyme. To this statement we may also add the step of P_i_ release, *i.e.*, whether the ATP binding step, the P_β_-O-P_γ_ γ-phosphorus–oxygen bond cleavage step, or the step of release of P_i_, any two of these steps, or all three steps in succession cause rotation of the γ-subunit in F_1_/F_1_F_O_ in the hydrolysis mode, or of the myosin head in myosin II/energy storage in the S-2 coiled coil of myosin II does not present problems for the torsional or RUT mechanism. Since each of these elementary steps of P_i_ binding, P_β_-O-P_γ_ bond formation, and ATP unbinding and release require energy as repeatedly enunciated by the torsional mechanism [1, 2, 10, 13, 17], the most interesting case for physiologically important ATP-hydrolyzing molecular machines such as conventional muscle myosin II (Sections 2–4), unconventional myosins, kinesins, and ncd (Section 6) is one where the reverse of each of these steps releases free energy in succession. It only remains to specify the quantum of energy released in each step. Here, I propose that this distribution of free energy released among the steps of ATP binding, P_β_-O-P_γ_ (γ-phosphorus–oxygen) bond cleavage, and P_i_ release is ∼9, ∼9, and ∼18 kJ/mol respectively, taking the standard Gibbs free energy of ATP hydrolysis at operating conditions of pH, pMg, and ionic strength, ΔG^0^′ as ∼36 kJ/mol. (The free energy released in each of these steps can be readily distributed in the same proportions if other values of ΔG^0^′ are assumed, and the unified theory is robust and does not require the particular value employed here; however, it is required of other values of ΔG^0^′ to ensure, after distribution of the ΔG^0^′ energy among the various steps, that energy competency for each step of the mechanism is satisfied). ADP release has the same triggering function in various physiological ATP-hydrolyzing molecular motors of the myosin and kinesin family (e.g. it unblocks the kink/localized strain at the S-1–S-2 hinge of myosin II, and triggers the coiling back of the first few N-terminal heptads of S-2, which enables utilization of the stored energy of S-2 and subsequently leads to the initiation of the power strokes of myosin on actin about the S-1–S-2 hinge of myosin as the fulcrum), as specified earlier in minute detail [10]. In general, it can be said that energy has to be supplied from another source or some other interaction is required (e.g. with another subunit or agent) to unbind the bound MgADP from the enzyme. However, it should be understood that in the case of *in vitro* ATP hydrolysis by F_1_-ATPase, when the special phenomenon of nucleotide exchange of bound ADP with medium ATP in site 2 (L-site) is operative (Section 2.4.3), or if ATP binds to one catalytic site and ADP is released from another catalytic site in the α_3_β_3_γ subcomplex of F_1_ then, since ADP release can cause distortions in the catalytic site during its release [10] and since there are interactions of the catalytic site with γ, there is no reason why ADP release cannot cause some rotation of the γ-subunit. In other words, the ADP-ATP nucleotide exchange from a single catalytic site, or the ADP release from a catalytic site, together with ATP binding to a different catalytic site, can drive the ∼80° sub-step of γ-rotation, in agreement with a recent proposal from sophisticated single molecule experiments on the α_3_β_3_γ subcomplex of F_1_ [30]. It should also be clearly understood that the binding energy of MgATP to myosin head is not ∼9 kJ/mol but rather it is ∼9 kJ/mol *larger* than the interaction energy of the actomyosin bond after the power stroke, because the MgATP binding energy to the myosin head is used to break the myosin-actin bond and the surplus (balance) of ∼9 kJ/mol is released and is available to cause conformational changes in myosin II, or be stored. The part of the free energy of binding of Mg-nucleotide to the enzyme that helps break the actin-myosin/F_1_ β-ɛ interactions, when added to the standard free energy change upon ATP hydrolysis of ∼36 kJ/mol, yields the total ΔG′ change of ∼55 to ∼60 kJ/mol for the entire cycle. Ultimately, it is imperative that this ∼55 to ∼60 kJ/mol per ATP synthesized be provided by the redox machinery in mitochondria during oxidative phosphorylation or by light energy in chloroplasts during photophosphorylation. It should also be noted that such a redistribution of free energies and such a mechanistic description in the ATP hydrolysis mode by the RUT energy storage mechanism is consistent with the description in the ATP synthesis mode by the torsional mechanism.

### 2.2. Contradictory Assumptions and Gross Inconsistencies among Previous Models as Seen from the Viewpoint of the Unified Theory

Thus, to arrive at a unified thermodynamic theory of the ATP catalytic cycle, it was necessary to specify the energetics of each step of binding, bond breaking and ligand release. This has been specified in Section 2.1. Of course, such an apportioning of energy release among the steps of ATP binding, hydrolysis and inorganic phosphate release (from being bound on the enzyme and removal to infinity, e.g. by release to the medium) contradicts existing theories in bioenergetics such as the binding change mechanism, which claims that the useful work is due to the substrate ATP binding energy released in the binding step. It should also be observed that theories in motility, such as the lever arm mechanism, attribute the power stroke to be primarily due to product phosphate/ADP release, which is completely different from that postulated by the binding change mechanism, as stressed earlier [10]. Why have there been these gross inconsistencies among previous models and how can the unified theory remove them?

### 2.3. Further Fundamental Differences between the Torsional Mechanism of ATP Synthesis and the Binding Change Mechanism

A major responsibility that has led to this state of affairs has to be taken by the binding change mechanism beginning ∼1970, which claimed as a central tenet that the actual chemical synthesis step in the making of ATP required no external energy, was in fact, gratis, and focused almost exclusively on binding energy of the MgADP/MgATP substrate and its transmission from one catalytic site to other catalytic sites of the enzyme (cooperativity) where it was hypothesized to be utilized for other functions (e.g. product release). We have repeatedly pointed out the falsity of this and several other tenets of the binding change mechanism [1, 2, 10, 11, 13, 16, 17, 21, 29]. The mechanism is inconsistent with basic biophysical and structural data, such as the observation of tri-site catalysis in F_1_-ATPase by tryptophan fluorescence [31, 32], and the tri-site occupancy seen in the transition state by the Leslie-Walker structure of 2001 [33], as has already been discussed at great length [1, 2]. (Other discrepancies with basic biochemical data will be pointed out in Section 3.1). Firstly, such a tenet is contrary to the known principles of chemistry. For instance, in phosphate chemistry, hydrolysis of the terminal phosphate bond is known to have a standard Gibbs free energy of reaction of ∼ −9 to −10 kJ/mol (and not zero). This is one reason why it was stated that in the binding change mechanism, the chemistry of the ATP hydrolysis/cleavage reaction and chemical reaction-linked conformational changes have not been given the importance they deserve and that the proposal had not been cast in detailed molecular terms to permit a proper evaluation [10]. This is not to say that the binding change mechanism has incorporated the physics of bringing charged moieties together correctly. In fact, after bond cleavage, if only one of the products is charged, as in the well-known case of hydrolysis of phosphoglycerate, then the value of ΔG^0^′ remains ∼ −9 to −10 kJ/mol. However, if both the products are charged, as in the MgADP-P_i_ case, then the standard free energy change rises to ∼ −36 kJ/mol, because of the Coulombic repulsion between the charged moieties, in our case, MgADP and inorganic phosphate, and this potential energy can be stored/can perform useful work. Hence, in the reverse process of bringing one of these charged moieties from infinity to the final bond distance in ATP of approximately 0.3 nm, we need to expend ∼36 kJ/mol, as per the known principles of electromagnetic theory. This then is the estimate of potential energy/stored energy between the charges MgADP and P_i_ considered by the torsional mechanism.

The above analysis implies that hydrolysis of the terminal phosphate-oxygen bond in ATP plus the electrostatic repulsion energy of the charged MgADP and P_i_ products works out to be ∼9 + ∼27 = ∼36 kJ/mol. In the reverse ATP synthesis mode, this ∼36 kJ/mol has to be transmitted to the catalytic sites from a source of potential/stored energy within a subunit of the F_1_ portion of the ATP synthase, which in turn is transduced (and stored) from the electrochemical gradients of ions, which itself is derived from redox/light energy. According to the torsional mechanism, the source for bringing (or rather forcing) the negatively charged MgADP and inorganic phosphate to a final P-P distance in ATP of ∼0.3 nm from infinity is the stored torsional energy within the γ-subunit of ATP synthase. The free rotation of γ envisaged by the binding change mechanism is simply powerless to directly perform this key function. In fact, the envisaged free rotation of the older mechanism is properly classified as a (rotational) *kinetic* energy. The torsional strain in γ postulated by the torsional mechanism, which arises from the theory of elasticity, is completely different, and is properly classified as a form of (storable) *potential* energy, or more precisely, as *elastic strain energy*, or the *energy of elastic* (and in this particular case, *torsional*) *deformation*, which, being *conservative* in nature, can be conserved and stored, and, ideally, is capable of being fully recovered later. Thus the difference between the two mechanisms is absolutely fundamental. As has been recently pointed out on p. 2232 of Ref. [10], when the γ shaft (whose movement is not continuous but discrete) slows down, the rotational kinetic energy will be thermalized and dissipated as heat, and not converted to useful external work or stored energy, *i.e.*, the rotational kinetic energy of the binding change mechanism, or of the so-called “rotational catalysis” has a *dissipative* character. Other fundamental differences have already been summarized in a tabular column on pages 132–133 of Ref. [2].

A major achievement of the torsional mechanism of ATP synthesis appears to lie in the fact that it dealt with and proposed unprecedented details of energy transduction in the ATP synthesis mode as far back as ten years ago [1, 2, 10–29], even though the vast majority of the experimental data then was (and still continues to be) in the hydrolysis mode collected without the membrane-bound F_O_ portion of the synthase and in the absence of the electrochemical ion gradients that drive the ATP synthesis process. Another key difference right from inception is between cooperativity, fundamental to the binding change mechanism, and asymmetry, which is fundamental to the torsional mechanism [1, 2, 13, 16–18]. The fact that the first molecule of substrate binds very tightly and with the highest affinity to a catalytic site of F_1_, while the second molecule of substrate binds with lowered affinity to a second catalytic site, and the third substrate molecule only binds with a very low affinity to a third catalytic site is attributed to a negative cooperativity of binding by the binding change mechanism. The torsional mechanism explains this experimental finding as arising from the asymmetric interactions of the catalytic sites with the single copy γ- and ɛ-subunits [1, 2, 16–19]. Thus, one of the catalytic sites interacts strongly with the ɛ-subunit, another catalytic site interacts with the γ-subunit while a third catalytic site interacts neither with γ nor with ɛ. Thus, according to the torsional mechanism, the catalytic site interacting neither with γ nor with ɛ binds substrate most tightly (site 1), the site interacting with γ binds substrate with intermediate affinity (site 2), and the site interacting strongly with the ɛ-subunit is the site of lowest affinity (site 3). Thus the observation of differing nucleotide affinities of the three catalytic sites does not have anything to do with a negative cooperativity of binding but, in stark contrast, arises from the asymmetric interactions of the catalytic sites with the single copy subunits of the F_1_ portion of the ATP synthase [1, 2, 16–19, 29]. According to positive catalytic cooperativity in the binding change mechanism, binding of the first molecule of substrate to the catalytic site yields only a slow uni-site catalysis rate of ATP synthesis/hydrolysis and increased substrate concentration leads to occupation of a second site which increases the catalysis rate constant and the enzyme reaches maximal activity due to a positive catalytic cooperativity among catalytic sites (which is further hypothesized by the binding change mechanism to operate *simultaneously* with the negative cooperativity of binding among the catalytic sites). However, according to the torsional mechanism, uni-site catalysis is a non-physiological mode of operation of the synthase, and the enzyme conformation with two sites filled is the resting (ground) state of the enzyme. Rotation and physiological steady-state ATP synthesis then only occurs when all three catalytic sites are occupied by bound Mg-nucleotide. (Thus, no continuous rotation or steady-state ATP synthesis or hydrolysis occurs in uni-site or bi-site modes of catalysis by F_1_F_O_ and the enzyme only functions in a steady state in the single mode of tri-site catalysis when all three catalytic sites are filled with bound Mg-nucleotide). Since the enzyme functions only in the tri-site mode, each substrate molecule enters and binds to the enzyme in the same (unchanging) state in each catalytic cycle, and hence there can be no question of change in catalysis rate constant with substrate concentration (and transition in the number of sites filled from one to two) in the physiological steady-state mode of operation, and hence we could not conceive of positive catalytic cooperativity in this mode of operation. These mechanistic aspects have been analyzed earlier [13, 16–19] and also discussed in previous reviews [1, 2, 29].

Finally, analysis of a general kinetic scheme of steady-state ATP hydrolysis by F_1_/F_1_F_O_ (Section 2.6) suggests the occurrence of *competitive inhibition* in F_1_-ATPase by MgADP as the inhibitor in the hydrolysis mode, which is consistent with the known property of MgADP as a competitive inhibitor in ATP hydrolysis (and also with the occurrence of “MgADP inhibition” due to its trapping in a catalytic site) [1–3]. This has important mechanistic implications because it imposes an order on binding and release events taking place on the enzyme, *i.e.* product MgADP release must precede substrate MgATP binding during steady-state hydrolysis, in contrast to the binding change mechanism which requires that substrate MgATP binding precede product MgADP release or be simultaneous with it in the hydrolysis mode in order that cooperativity and signal transmission from one β catalytic site (via an intervening α-site) to another β catalytic site can occur. Since the analysis (Section 2.6) is based on a general kinetic scheme that is applicable to all mechanisms, the above implication is valid irrespective of the specific mechanism.

### 2.4. Further Development of the Torsional Mechanism of ATP Synthesis/Hydrolysis, the Rotation-Uncoiling-Tilt (RUT) Energy Storage Mechanism of Muscle Contraction and the Unified Theory

#### 2.4.1. Complete Details of Quantized Release and Utilization of Energy in the Synthesis Mode and its Mechanistic Implications

The mechanism by which the electrochemical gradient of protons and anions is transduced to the torsional energy in the γ-subunit of ATP synthase has already been elaborated within the torsional mechanism [1, 2, 10–13, 15, 19]. I now further propose that of the total torsional energy stored in γ (estimated as ∼54 kJ/mol in the entire process, though not all the ∼54 kJ/mol is present as stored torsional energy at an instant of time, as described further) the required quanta of energy are released in stages, distributed and used. First, upon ion translocation in the membrane-bound F_O_ portion of ATP synthase, as the bottom of γ attempts to rotate counterclockwise (viewed from the F_1_ side) while the top of γ is stationary, ∼9 kJ/mol torsional energy of γ is used to help break the ɛ-β_E_ interactions by adding to the binding energy of MgADP in the β_E/C_ catalytic site in the F_1_ portion of ATP synthase. Thus, in the O (open and empty) site, MgADP binds with a binding energy of ∼27 kJ/mol and as the site closes, a new intermediate closed site, C is created where the MgADP binds tighter, with a binding energy of ∼35 kJ/mol. Thus C contains tightly bound MgADP. These changes occur during the 0–30° counterclockwise rotation (viewed from F_1_) of the bottom of the γ-subunit [1, 2, 17, 18] (taking the number of subunits in the c-oligomer as 12 primarily for *pedagogical* reasons and for ease of explanation of a complex mechanism, but the molecular mechanism readily works for other numbers, including 10, the number employed for thermodynamic analysis in Section 4.3; in general the angle rotated in each step would be 360°/n, where n is the number of c-subunits). Another ∼9 kJ/mol of torsional energy of γ is released and used to create the site for inorganic phosphate binding. This happens during the 30–60° counterclockwise rotation step of the bottom of the γ-subunit, with the top of γ stationary. Between 60–90° counterclockwise rotation of the bottom of γ while the top of γ is stationary and does not rotate, P_i_ binds in the newly created site/pocket and now the occupancy of the site, let us call it C′, is MgADP + P_i_. In the C′ conformation of the catalytic site, both the substrates MgADP and P_i_ are bound; however, the β-phosphate of MgADP and the inorganic phosphate are too far apart to interact and form the terminal phosphorus-oxygen bond, as clearly inferred from the structure of the half-closed conformation with a sulfate group mimicking the phosphate [33], *i.e.* they are not in an *activated* state for nucleophilic attack and subsequent bond formation. Incidentally, it should be noted that the binding change mechanism only incorporates O (open), L (loose) and T (tight) conformations of the β catalytic sites, and such intermediate C (closed) or C′ (closed) conformations of the catalytic sites between the O and L conformations appear nowhere in the mechanism, as pointed out earlier as a defect [1, 2, 17]. However, a C′-site has been clearly visualized in the X-ray structure of 2001, as mentioned above [33], and designated HC (for half-closed), but this HC nomenclature is with reference to the open O-site, and a quarter-closed, half-closed, or three-quarter-closed etc. site can always be termed a closed (C) site with respect to the open (O) site. Furthermore, in the torsional mechanism, we had predicted the occurrence of the new intermediate closed (C) site with respect to the open or empty (O or E) site [16–19] several years before the solution of the crystal structure [33]. To continue, P_i_ binding releases ∼9 kJ/mol energy, which affects (lowers) the threshold torsional strain in γ at ∼90° angular position, and thus helps the top of γ to rotate and release the stored torsional energy in the next step. Thus, finally, during the 90–120° step of the counterclockwise rotation of the bottom of γ, the accumulated torsional strain in γ crosses the threshold and the constraints present at the top of the γ-shaft (due to the interactions of the top of the γ-subunit with the β catalytic sites) are broken and the top of the γ-subunit now rotates counterclockwise (seen from the F_1_ side) in a single step from 0–120°, and the remaining ∼36 kJ/mol of torsional energy stored in γ is released, transmitted to the β catalytic sites and used to convert the C′-site to the L-site and the L-site to the T-site [1, 2, 10–13, 16–18, 29]. The L-site contains bound MgADP.P_i_ with the MgADP–O^−^ and the HPO_4_^2−^ now *activated* for nucleophilic attack, which occurs in the next conformational change (L to T), leading to the formation of the transition state and further to terminal phosphorus-oxygen bond formation in ATP, and the T-site contains tightly bound MgATP waiting to be unbound and released during the subsequent T → O transition of the catalytic site due to interaction of the ɛ-subunit with the T-site, converting it to an O-site. During the C′ → L transition, MgADP concomitantly binds in L with a reduced binding energy (by ∼9 kJ/mol), compared to its binding energy in the C′-site, such that MgADP is bound in L with a binding energy of ∼27 kJ/mol, while MgATP concomitantly binds tighter in T by ∼9 kJ/mol (compared to the binding of MgADP.P_i_ in L) during the L → T transition, and the binding energy of the MgATP in the T-site works out to be ∼45 kJ/mol. The critical role of Mg^2+^ in this catalysis has already been described in detail [1, 2, 17–19]. Approximately 18 kJ/mol is distributed and employed for the C′ to L transition and the activation process (the P-P distance is reduced from infinity earlier, before phosphate binds, to ∼0.6 nm in L), and another ∼18 kJ/mol is utilized to force the L to T transition (there is a reduction in the P-P distance from ∼0.6 nm in the L-site (β_TP-like_) to the transition state distance of ∼0.4 nm, and then a compression of the transition state to a P-P distance of ∼0.3 nm in the T-site (β_DP-like_), the bond distance in ATP). During the final bond formation step, as the P-P distance reduces from ∼0.4 to ∼0.3 nm, the MgATP concomitantly becomes more tightly bound in the β_DP-like_ catalytic site by ∼9 kJ/mol than MgADP.P_i_ was bound in the β_TP-like_ catalytic site. We have thus arrived at a diametrically opposite view from the binding change mechanism in which the making of the ATP was a trivial thing that merited no description, and the energy quantum required for the process was dismissed as inconsequential. In contrast, according to the torsional mechanism, the chemical synthesis of ATP by conformational changes is an exquisite process in which everything cannot be performed in one step but requires a number of ordered, sequential steps with quantization of energy as detailed above.

**Figure 1.**
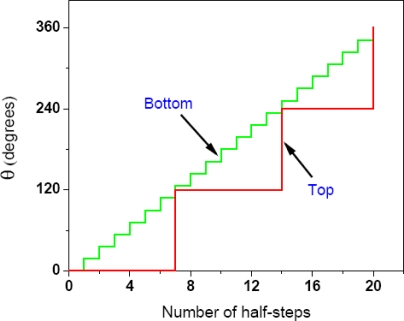
Torsional mechanism for F_1_F_O_-ATP synthase with symmetry mismatch. The diagram is drawn for a ring of ten c-subunits (i.e., n=10) and half-steps of 360°/(2n). A symmetry mismatch between c and γ rotation of 6° after 120°, and 12° after 240° [for half-steps of 360°/(2n)] is found. After 360°, it is found that the symmetry mismatch becomes zero.

**Figure 2.**
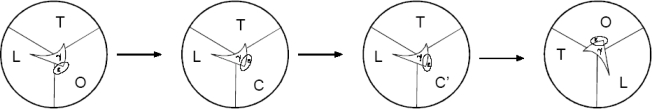
The torsional mechanism of energy transduction and ATP synthesis in the F_1_ portion of ATP synthase. The bound nucleotide occupancies of the catalytic sites during ATP synthesis are: no bound nucleotide in O, MgADP in C, MgADP + P_i_ in C′, MgADP.P_i_ in L, and MgATP in T. The diagram is drawn for one-third of the complete enzymatic cycle. Note that mismatch is removed only after the complete enzymatic cycle.

Before concluding this section, it should be re-emphasized that the torsional mechanism of ATP synthesis readily works for other values of H^+^/ATP stoichiometries and for different numbers of c-subunits in the c-oligomer of ATP synthase, for instance, ten, the number favored in Section 4.3 (Figures 1, 2). For a c_10_ ring, three ATP molecules will be synthesized in each 360° revolution of the c_10_ oligomer, and ten protons will be required, *i.e.* an average of 3.33 protons per ATP synthesized; thus the c_10_ stoichiometry of the c-ring dictates a nonintegral H^+^/ATP ratio. Each step of rotation in F_O_ will then measure 36° and each sub-step, 18° (Figure 1). This means that, starting from a strain-free resting state at an angular position of 0°, the c-oligomer and the bottom of the γ-subunit will rotate in 36° steps (instead of the 30° steps used in our simplified analysis in this section in order to facilitate easier understanding of a complex and dynamic mechanism), and the short pauses or dwells will be at 36°, 72°, 108° and 144° (= 24°) (for synthesizing the first ATP molecule), at 144° (= 24°), 180° (= 60°), 216° (= 96°) and 252° (= 132° = 12°) (for synthesizing the second ATP molecule), and 252° (= 132° = 12°), 288° (= 48°), 324° (= 84°) and 360° (= 120° = 0°, back to the resting state after a full revolution) (for synthesizing the third ATP molecule). Thus, four protons will be required to make the first ATP molecule and three protons each to synthesize the second and the third ATP molecules in one complete 360° revolution, *i.e.* on the average, 3.33 protons will be needed to synthesize one ATP molecule, or H^+^/ATP = 3.33. For a 10-subunit c-ring, ∼180 meV of energy from the elementary ion translocation events in the F_O_ portion of ATP synthase produces each rotation step of 36° {*i.e.*, each elementary act of proton/anion binding and unbinding creates a local electrical potential of ∼45 mV in the access half-channels at the a–c interface in F_O_ (Sections 5.1–5.5)} and stores ∼16.2–16.5 kJ/mol as torsional energy in γ. An input of ∼9 kJ/mol from the torsional energy stored in the γ-subunit is required to help MgADP bind tightly in the C conformation (this section), and according to the torsional mechanism [1, 2, 16–19], because *the top of γ always rotates in 120° steps (irrespective of the number of c-subunits in the c-oligomer)*, the γ-subunit possesses ∼12 kJ/mol torsional energy at the pause or dwell at an angular position of 144° (= 24°), sufficient to help MgADP to bind tightly, and ∼6 kJ/mol at the pause or dwell at 252° (= 132° = 12°), which can be readily increased upon γ rotation and storage of torsional energy and made sufficient (to ∼9 kJ/mol) and then donated to enable tight MgADP binding in C, as described in this section and earlier [1, 2, 16–19]. Further, P_i_ can readily bind following the formation of a P_i_-binding pocket by γ rotation after a single step of 36°. Hence three steps in the catalytic cycle at the bottom of the γ shaft at the end of which MgADP binds, P_i_ binds, and the contacts at the top of the γ shaft are broken (following which the top of γ rotates in a single 120° step and ∼36 kJ/mol torsional energy is released) respectively are minimally required to allow all the events described in this section to occur. In Figure 1, the torsional mechanism with symmetry mismatch and sub-steps of 18° for a cring with ten c subunits is illustrated. Thus, in conclusion, *the mechanism works whether there is symmetry or there is symmetry mismatch between F_1_ and F_O_*. It is often believed that symmetry mismatch between the catalytic and rotor domains is a fundamental intrinsic feature of F-type ATPases [33] and that elasticity in the central or peripheral stalk is present only to permit a mismatched symmetry to operate. On the other hand, according to the torsional mechanism, symmetry mismatch between F_1_/F_O_ is not obligatory, and the property of torsional elasticity of the central stalk is a fundamental intrinsic structural and mechanistic feature of F-type ATPases and torsional energy storage in the γ-subunit of ATP synthases has a central role in function, *i.e.*, in synthesizing ATP, irrespective of whether there is F_1_/F_O_ symmetry mismatch or no mismatch. This is also evidenced from the history of the development of the torsional mechanism [1, 2, 10–29], which was proposed *before* the presence of a mismatched symmetry in ATP synthases from certain sources was revealed by structural studies (see Section 4.3). This central role of the torsional strain in the γ-subunit of F_1_F_O_-ATP synthase has been highlighted and repeatedly emphasized, and has been captured in the name of the mechanism itself.

Finally, the stoichiometry of the c-ring is not a variable but is an intrinsic, structural property of the c-subunits of the F_1_F_O_-ATP synthase from a particular source. The exact value of the local electrical potential created by ion translocation at the sites in F_O_ will depend on the *local* electrical conductivity in the F_O_ portion of the ATP synthase in the energy-transducing membrane. Since the energy required to synthesize a molecule of ATP is constant (in contrast to the view originating from observed c-ring stoichiometries that the energy required to make an ATP molecule is variable [33]), the lower the value of the local electrical potential, the higher the number of c-subunits required in the c-oligomer has to be, and the higher will be the H^+^/ATP ratio (and the lower the efficiency (Section 4.3), other things being the same). The variability in the stoichiometry of ATP synthases from different sources can then be readily explained as arising from the different electrical properties of the energy-transducing membranes of synthases from different sources. Thus, the variable number of c-subunits in the c-oligomer can be viewed as adaptation mechanisms and have fascinating evolutionary implications, discussion of which however is beyond the scope of this paper.

#### 2.4.2. Quantized Release and Utilization of Energy in the Hydrolysis Mode and Its Mechanistic Implications for Muscle Contraction

Once ATP synthesis has been solved in detail, as given above, the ATP hydrolysis case can be readily worked out. From the above discussion, the energy transduction does indeed occur at the hydrolysis bond cleavage step [10], but whether all or only part of the post-hydrolysis Coulombic repulsion energy between the products is released at the hydrolysis step itself or remains stored in the form of potential energy and is only released at a later step (e.g. upon release of phosphate to the medium) depends upon whether the γ-phosphate can move away from the MgADP to infinity at the hydrolysis bond cleavage step, as tacitly assumed [2, 10, 11], or not. The former assumption need not be true, as pointed out in a recent prescient and most valuable suggestion by Ross [34]. Thus, if MgATP is *tightly bound* to the catalytic site of the enzyme, ATP hydrolysis can take place on the enzyme, but the P_i_ cannot move away from the MgADP, even though there exists Coulombic charge repulsion between the two species. Hence, reduction in Coulombic repulsion cannot take place, and the potential energy between the charges cannot be converted into mechanical work, but remains stored as potential energy *until the binding is reduced*. This reduction of binding can occur at a later step (e.g., of P_i_ release) and the electrostatic repulsion energy of hydrolysis can thus be stored/used for performing work at a later instant of time. Hence the source of energy is indeed the hydrolysis step, but the release of energy is distributed over several steps of the enzymatic cycle. This change is readily made on pages 2223 and 2228 of Ref. [10] by substituting the word “during ATP hydrolysis” with “during ATP hydrolysis and later” or more specifically in the context of the present paper by “during ATP hydrolysis and subsequently upon P_i_ release.” Added to this the fact that the available balance of MgATP binding free energy to myosin (estimated as ∼9 kJ/mol for muscle myosin) over the interaction energy of actin-myosin also causes rotation of myosin head and can be stored in S-2 implies that the binding step, hydrolysis step *per se*, and the potential energy between the charged products upon ATP hydrolysis that is released upon phosphate release to the medium (*i.e.* to infinity), all contribute to the high-energy state of the S-2 coiled coil of myosin II, and thus the free energy release is distributed over these three steps of the ATP cycle (∼9 + ∼9 + 18 kJ/mol respectively) and stored/locked in a high free energy (by ∼36 kJ/mol) uncoiled nonequilibrium conformational state of the S-2 coiled coil of myosin II and is subsequently used to produce the power strokes of the two myosin heads on actin about the S-1–S-2 hinge as the fulcrum as described earlier [2, 10, 11, 14]. The paragraph in column 2 of p. 2229 of Ref. [10] needs to be modified in the light of the insights of this section, which had also been alluded to on p. 2232 of Ref. [10] and further in Section 2.1 of this paper.

These ideas take us beyond the lever arm model of muscle contraction from the point of view of the driving forces for the power strokes, the pressing need to consider both S-1 and S-2 together as forming the muscle “crossbridge” (unlike S-1 alone currently in the lever arm model), the crucial function of energy storage in the S-2 coiled coil, and the key role of hydrophobic interactions (the hydrophobic residues of the coiled coil are forced to intrude into a region of liquid water, thereby storing the free energy of mechanical torque/elastic deformation in a high free energy state of the submolecular coiled coil element of myosin II, with the thermodynamic propensity of water to repel the hydrophobic residues of S-2 and regain the stabler low free energy resting state of the S-2 coiled coil a primary driving force for muscle contraction). Finally, it should be noted that the fulcrum of the power stroke was postulated to be the actin-myosin attachment site by the swinging crossbridge model, and this view prevailed for almost three decades from ∼1955 - ∼1985. The lever arm model postulated the further distal movement of the fulcrum from the actomyosin site to the junction of the catalytic and regulatory domains within the myosin head, and this view has held sway for the past two decades from ∼1985 to date. According to the RUT energy storage mechanism of muscle contraction, there is a two-way communication of energy along the helical backbone structure of myosin II (from S-1 to S-2 and then back again from S-2 to the actomyosin) and the S-1–S-2 hinge is the true fulcrum of the power strokes of myosin heads on actin executed during the backward release of stored energy. Hence the location of the hinge about which the power strokes occur needs to be moved further distally once more, and it can be stated with confidence that here, after five decades, it will finally have found its abode of peace. Other generic differences between the RUT energy storage mechanism and other models of muscle contraction have been covered in much detail in earlier papers (especially pp. 2227–2229 of Ref. [10], pp. 160–164 of Ref. [2], and p. 82 of Ref. [14]).

#### 2.4.3. Complete Details of Quantized Release and Utilization of Energy in the Hydrolysis Mode and its Mechanistic Implications for F_1_-ATPase

After the above analysis, complete details of the discrete, quantized nature of energy release in the ATP hydrolysis process and especially by F_1_-ATPase *in vitro* can be better understood. Upon cleavage of the terminal phosphorus-oxygen bond in ATP, due to the T → L *conformational transition* of the β catalytic site caused by ∼80–90° rotation of the γ-subunit, there is a change in the P-P distance from ∼0.3 nm to a distance of ∼0.4 nm in the transition state, and ∼9 kJ/mol of free energy is released. (Simultaneously, upon crossing the transition state, the binding energy of the MgADP in the new L conformation is reduced by ∼ 9 kJ/mol compared to the binding energy of MgATP in the T conformation). The free energy released is used to weaken the binding of P_i_ to the enzyme catalytic site by ∼9 kJ/mol. This reduction of binding of P_i_ leads to a reduction of Coulombic repulsion between MgADP and P_i_, and the phosphate moves apart from MgADP to an intermediate separation between them, which is predicted to be ∼0.6 nm, and involves a Coulombic energy change of ∼9 kJ/mol. This ∼9 kJ/mol energy released can be stored (e.g. in the S-2 region of myosin), or be used to weaken some other interaction (e.g. ɛ-β_E_ in F_1_-ATPase), or can cause other conformational changes. Upon actin/microtubule interaction or interaction of the γ-subunit with the catalytic site in F_1_-ATPase, P_i_ is released to the medium (this occurs *after* clockwise rotation of γ (looking from the F_1_ side) by ∼80–90° during the P_i_-release waiting dwell of ∼1 ms duration which follows the ∼1 ms long catalytic dwell in which hydrolysis occurs [30] and, due to the further reduction in electrostatic potential as the distance between the charges increases from ∼0.6 nm to infinity, a further free energy release of ∼18 kJ/mol takes place. (All of these discrete, quantized energy release events occur in a medium dielectric constant of ∼25 typically found in a non-membranous protein fold). The ∼18 kJ/mol released is further stored as an uncoiled state of S-2 in myosin II, or causes clockwise rotation of γ-subunit (looking from the F_1_-side) by approximately 40° (for which it has the requisite energy competency). Thus, previous work (p. 138 and the entry in the right hand side column on page 133 of Ref. [2], and p. 12 and the Legend to Figure 8 on p. 13 of Ref. [11]) needs to be modified to ascribe only the ∼40° sub-step to the ATP hydrolysis energy, and not the entire 120°, and the ∼80° sub-step to another source (given in the next few lines). I further propose for the first time (and this is genuinely and startlingly novel) that the sub-step of ∼80° clockwise rotation (looking from F_1_) of the γ-subunit in the *in vitro* experiments on F_1_-ATPase or F_1_F_O_ occurs due to *ADP-ATP nucleotide exchange* (*i.e.* exchange of the bound ADP in the catalytic site with medium ATP) in site 2 (β_TP_). This ADP-ATP exchange in site 2 (L-site or β_TP_), *i.e.* the release of ADP and the binding of ATP to the site with intermediate affinity in F_1_, contributes a free energy of at least ∼35 kJ/mol in the catalytic site which is energetically competent to cause the ∼80° rotation of the γ-subunit. It must be especially emphasized that in the ATP synthesis mode *in vivo*, at the physiological nucleotide concentrations in the matrix/stroma in mitochondria/chloroplasts, such a nucleotide exchange mechanism from site 2, or for that matter from any of the catalytic sites of the ATP synthase enzyme, does not occur. This is thus a major difference between the *in vivo* synthesis and laboratory *in vitro* hydrolysis modes of functioning of the enzyme. This is also a major reason why *the molecular mechanism of one mode is not an exact reversal of the other mode*. Further, the MgADP inhibition that has been routinely observed and documented in V_max_ ATP hydrolysis has never been observed during physiological ATP synthesis, again pointing to a lack of exact reversal of the two modes. As the unified theory shows, the dwells and sub-steps of ATP synthesis caused by the ion gradients are *different* from, and do not mirror, the dwells and sub-steps of ATP hydrolysis by F_1_-ATPase. This then is the answer to a question posed recently [35]. (It has been repeatedly stressed that the mechanisms, though macroscopically irreversible [1, 2, 12, 13, 29], are microscopically reversible [2, 11]). In fact, I suggest that, once such a nucleotide exchange from the loose site (site 2) is not operative in F_1_-ATPase, since the tight site (site 1) contains tightly bound, *non-exchangeable* MgATP and the open site (site 3), which is interacting with the ɛ-subunit according to a key proposal of the torsional mechanism [1, 2, 16–19], is empty of bound nucleotide in the resting state (true ground state) of the enzyme [1, 17–19], or contains bound Mg-nucleotide whose binding energy is not even sufficient to break the ɛ-β_E_ interactions, let alone break the interactions *and also rotate γ*, there would be no agent to drive the ∼80° rotation sub-step of γ. This ∼80° rotation of the γ-subunit is the *initiation step* and a prerequisite for causing the change in conformation of the tight site to the loose site that enables the bound MgATP to hydrolyze to MgADP.P_i_ in the new loose site and hence ATP hydrolysis cannot occur. Thus, during ATP synthesis under physiological conditions in mitochondria and chloroplasts *in vivo*, ATP hydrolysis cannot take place. Finally, in the absence of the ɛ-subunit and owing to the crucial role of this subunit in energy coupling according to the torsional mechanism [1, 2, 16–19], experiments with the α_3_β_3_γ subcomplex of F_1_, popularly used in single molecule hydrolysis studies (and inappropriately called “F_1_” [30]) can lead to artefactual mechanistic results since it is a different system from the complete F_1_/F_1_F_O_ (containing ɛ). For instance, in the α_3_β_3_γ subcomplex of F_1_, even the identity of catalytic sites is altered compared to intact F_1_ or F_1_F_O_, and further, the kinetic/steric and energetic barrier due to ɛ-β_E_ interactions (which is a key novel proposal of the torsional mechanism) is removed due to the absence of the ɛ-subunit itself in the subcomplex. Thus, in the absence of the ɛ-subunit, the O-site exhibits properties, especially of nucleotide binding affinity, akin to that of the C-site and is now the new site 2, intermediate in affinity between L and T. In the absence of ɛ, binding of MgATP to such a C-site together with MgADP release from the L-site can drive rotation of the γ-subunit by ∼80°. Hence, in the α_3_β_3_γ (minus ɛ) subcomplex of F_1_, the hydrolysis mechanism is indeed bi-site, as proposed recently [30]. Thus, in the α_3_β_3_γ subcomplex of F_1_, *due to the absence of the ɛ-subunit*, the rotation of γ takes place with only two catalytic sites occupied by bound nucleotide and does not require filling of the third catalytic site, and the MgATP bound in C at 0° will be released as MgADP from L, *i.e.* upon the sequential C → T and T → L transitions, after (120° + 120°) = 240° angular rotation of γ. However, the fact that the catalysis is bi-site in experiments on α_3_β_3_γ has no bearing on, and cannot be extrapolated to what it would be in hydrolysis by F_1_ (especially given the crucial role of the ɛ-subunit discussed above and in earlier works [1, 2, 16–19]) let alone synthesis by the entire F_1_F_O_, which become hierarchical super-systems. This answers yet another question posed recently in the literature [35]. In contrast, a true understanding of the molecular mechanism of the physiologically meaningful ATP synthesis process by F_1_F_O_-ATP synthase enables us to understand also the mechanism of the ATP hydrolysis phenomenon by F_1_, or even by α_3_β_3_γ, as these are all sub-systems of F_1_F_O_.

### 2.5. Removal of the Various Inconsistencies in Previous Models by the Unified Theory

#### 2.5.1. Models of Muscle Contraction and Motility

Now the various apparently contradictory assumptions and inconsistencies between the majority views of researchers in bioenergetics and motility can be readily reconciled. In current models of muscle contraction such as the lever arm model, force generation and movement is considered to be directly coupled to the release of bound ligands/nucleotides (e.g. “phosphate/ADP release does work”) while according to the bioenergetics literature, release of bound nucleotides requires redox/light energy or the energy of ion gradients, which is quite the opposite assumption. First of all, production of the elementary contractile force and movement is not directly coupled to release of ligands, because the energy of MgATP binding, MgATP hydrolysis and P_i_ release has to be first stored in a nonequilibrium conformational state in a localized region of myosin before it can be released and lead to force production and movement. But such statements are routinely made because in the conventional view, it is not deemed necessary to include energy storage in muscle myosin, which according to the RUT energy storage mechanism of muscle contraction is a central aspect of the problem. Secondly, it is not as if kicking off phosphate/ADP does work, which may be misleading (in fact, MgADP release does indeed require energy from another source). As shown by the reactions of phosphate hydrolysis (e.g. phosphoglycerate mentioned above), if only one of the products is charged and the other is not, then release of the ligands cannot perform useful work. *In reality, the energy is still the energy of ATP hydrolysis, which remained stored as potential energy* (*i.e.* the energy of Coulombic repulsion) between the two charged products, ADP and phosphate, until reduction of binding, which allowed the phosphate to move away from the ADP. It is, in fact, ∼half of the total ATP hydrolysis energy, whose release has been *delayed* in time and allowed to be distributed in another elementary step. For conventional muscle myosin, since all the ∼36 kJ/mol is stored in a single coiled coil, this distribution of the energy over other elementary steps of the catalytic cycle may appear puzzling, but such distribution of free energy over other elementary steps of the cycle is absolutely necessary for proper functioning of other large classes of molecular machines which are double-headed and require processive motion on a microtubule/actin track, such as kinesins and unconventional myosins (Section 6). This is because ∼18 kJ/mol has to be made available after a time lag (upon phosphate release, which ensures that the first and now leading head which had stepped forward by ∼9 kJ/mol of MgATP binding energy + ∼9 kJ/mol of hydrolysis energy is bound tightly to the track and hence cannot be released except by MgATP binding at a subsequent stage in the enzymatic cycle) and is used to move the second rear head, which is bound loosely to the track, forward past the tightly bound front head as per the rotation-twist energy storage mechanism for processive molecular motors [10, 11]. If the entire energy had been released at the time of hydrolysis, then the first head would move double the distance, or the energy would be dissipated; in any case (even if somehow this energy were conserved), there would be no energy source left to move the second head forward in an *asymmetric* way on the other side of the track (compared to the first head), and the processive movement of the molecular machine on its microtubule/actin track would be arrested. Moreover such discrete, step-wise energy release is at the heart of biologically designed molecular machines due to the quantized release process of ATP hydrolysis described here. Only through an in-depth analysis, and a true understanding of the entire process of energy transduction, accumulation, storage, release, transmission, distribution and utilization in a unified way, as in this work and in our earlier works [1, 2, 10–29], are we able to evaluate the various statements made, and lend them a logical and intellectually satisfying interpretation.

#### 2.5.2. Models in Bioenergetics

This is not to say that researchers in bioenergetics have not committed errors. A cornerstone of the binding change mechanism is the postulate that ATP forms reversibly and spontaneously from ADP and P_i_ in a catalytic site with an equilibrium constant value, K_eq_ ∼1, *i.e.*, ΔG^0^∼0 [7]. In fact, according to Boyerean dynamic reversibility, one can go back and forth between ADP.P_i_ and ATP (*i.e.* form the bond and break it) repeatedly, without requiring any energy input, and that this occurs as many as 400 times before tightly bound ATP is released from the catalytic site. This concept is false, as repeatedly point out by us [1, 2, 11, 13, 17], and arose from an inept interpretation of oxygen exchange data, in which the fundamental error was made that the timescale on which oxygen exchange occurred was ignored (and any change in the extent of oxygen exchange was attributed instead to an alteration in the value of the exchange rate constant, and not to the time available for exchange, which should have changed with substrate concentration, but unfortunately this was not considered in the Boyerean equations), as revealed by a kinetic analysis from first principles [11]. Such a kinetic analysis revealed a perfect constancy of the rate constant of exchange over *five decades of substrate (MgATP) concentration* for mitochondrial F_1_-ATPase (Figure 7 of [11]). Even in an irreversible situation, when there is an energy trade-off or exchange, a redistribution such that free energy lost by one entity/subunit equals the free energy gained by another entity/subunit, there is a *net* free energy change of zero; but then this is not an equilibrium situation with K_eq_= 1. For illustration, let us apply the unified theory to the situation upon cleavage of the terminal P-O bond in ATP. The standard free energy change of reaction is ∼ −9 kJ/mol, and is conventionally given a negative sign. This energy is immediately used to weaken P_i_ binding to the site, *i.e.* the binding free energy of P_i_ to its site is now less negative (*i.e.* less stable) by ∼ +9 kJ/mol. The net change in standard state free energy is thus zero due to the free energy trade-off in the site, but this does not mean that it is a dynamic equilibrium situation and that the changes can move back and forth; in fact the free energy flow is strictly one-way during the hydrolysis process. During steady-state synthesis, the flow would be one-way in the reverse direction, and ∼ +9 kJ/mol of external energy will concomitantly lead to a tighter binding of MgATP to the site, *i.e.* a change of ∼ −9 kJ/mol, with a net standard free energy change of zero again. But this does not mean that “no external energy was required” (in fact 9 kJ/mol of external energy has been used), nor that this is an “equilibrium situation with K_eq_= 1.” This was a false interpretation of the binding change mechanism, and certainly it was not the only way to look at the energy transfer process, as explicitly revealed by the torsional mechanism and the unified theory, and the important arguments on p. 73 of Ref. [1] still retain their validity. Moreover, thermodynamic calculations based on experimentally measured site dissociation constants for MgADP/MgATP can further help adjudicate on this point (Section 3.1). The torsional mechanism of ATP synthesis has directed attention to an *irreversible* mode of operation of the ATP synthase [1, 2, 10–29]. Thus, a discrete, unidirectional rotation of the γ-subunit during steady-state synthesis or hydrolysis by ATP synthase is only possible due to the presence of a driving force in one direction. The driving forces in the synthesis mode are the concentration gradients of membrane-permeant ions, while ATP and its cleavage products and their release are the energy sources for reverse rotation in the hydrolysis mode, *i.e.* the agents/energy sources driving rotation of the γ-subunit are different in the two modes, and both driving forces or modes do not operate *simultaneously* in the same enzyme molecule, as conceived by Boyerean dynamic reversibility in particular, and by reversible catalysis in general. Thus, in steady-state operation, a single enzyme molecule either functions in the synthesis mode and the γ-subunit rotates in the counterclockwise sense (viewed from F_1_) or works in the hydrolysis mode in which the γ-subunit rotates continuously in the clockwise sense (viewed from F_1_), *in agreement with single molecule experiments*, and does not alternate from one mode to another as conceived by the binding change mechanism (where such a reversal is presumed to occur as many as 400 times before a single product molecule is released into the medium). This is due to the fact that the initial and boundary conditions of the macroscopic system especially in terms of permeant ion concentrations and substrate MgADP/MgATP concentrations are different in the two modes. Thus, ion binding and unbinding processes to/from their binding sites in the membrane-bound F_O_ portion of the ATP synthase along their concentration gradients drive steady-state ATP synthesis in counterclockwise ∼151°/∼30° sub-steps of the rotating elements, while concentration gradients of ADP (from a catalytic binding site in F_1_ to the medium) and ATP (from the medium to a catalytic binding site in F_1_) and subsequent ATP hydrolysis and P_i_ release to the medium drive the rotating elements in clockwise ∼80°/∼40° sub-steps (with *analogs* of ATP) or ∼90°/∼30° sub-steps (with ATP) during steady-state hydrolysis, as addressed in consummate detail in the unified theory.

As discussed above, the torsional mechanism of energy transduction and ATP synthesis, the RUT energy storage mechanism of muscle contraction, and the unified theory have directed attention to the importance of an irreversible mode of operation of the energy-transducing enzyme [1, 2, 10–29]. This does not mean that the enzyme cannot be reversed (in this sense of usage of the term “reversibility,” the ATP synthase enzyme is indeed *reversible*), but that due to the initial and boundary conditions prevalent in the *in vivo* system or imposed by the experimentalist on the system *in vitro* that ensure the presence of a driving force in one direction, the enzyme works in a single mode (synthesis or hydrolysis), until these initial and boundary conditions are altered.

In addition to the ∼9 kJ/mol released upon cleavage of the terminal P-O bond in ATP (discussed above), there is furthermore ∼9 + ∼18 kJ/mol, the latter although it is released upon phosphate release, is, in reality, a part of the hydrolysis potential energy, as discussed in Section 2.4.2. In the reverse mode, during physiological ATP synthesis, energy has to be supplied by the torsional energy of γ and used to convert the C′ catalytic site containing MgADP + P_i_ to the loose site containing MgADP.P_i_ (∼18 kJ/mol) and another ∼9 kJ/mol + ∼9 kJ/mol used during the L to T conformational transition of the catalytic site to form the transition state and compress the transition state respectively in order to reach the final P-P distance in ATP of ∼0.3 nm.

Over time, attempts have been made to modify the binding change mechanism, for instance to try and include the experimental fact of tri-site catalysis (as opposed to the bi-site catalysis of the original binding change mechanism), but without altering other fundamental tenets of the mechanism. First of all it is a matter of kudos that in a subject where there is much variance, the assignment of the structurally tight site (β_DP-like_) and the structurally loose site (β_TP_ or β_TP-like_) in the works of Walker and Leslie, Senior, and Nath are identical. It is a different matter that computational approaches like MD-simulations in the hands of several investigators and other methods have led to β_TP_ being assigned as the tight site [36–38]. We completely disagree with this latter assignment as evident from close structural inspection, magnesium coordination chemistry considerations, and calculations of the buried surface area at the catalytic interfaces. The order of conformations that a catalytic site passes through *during hydrolysis* is postulated by Menz *et al.* [33] to be:

(1) $$\beta_{\text{E}}\to ∼\beta_{\text{HC}}\to \beta_{\text{TP}}\to \beta_{\text{DP}}\to \beta_{\text{HC}}\to \beta_{\text{E}}$$

where the ∼ sign means that the state has not yet been structurally visualized. Note that the transition state has been excluded in the equation. Therefore, for *synthesis of ATP* they have to have:

(2) $$\beta_{\text{E}}\to \beta_{\text{HC}}\to \beta_{\text{DP}}\to \beta_{\text{TP}}\to \;∼\beta_{\text{HC}}\to \beta_{\text{E}}$$

The torsional mechanism had made 15 predictions for ATP synthesis by F_1_F_O_-ATP synthase, which were listed in 2002 (pp. 79–80 of Ref. [1]; see also pp. 132–133 of Ref. [2]). We still stand by these predictions made for the synthesis mode. The order of conformations that we had predicted is in contradiction with the order postulated by Menz *et al.* (Eq. (2)), though our predictions of the order of conformations of a β catalytic site are in consonance with Senior [3]. However, Senior and colleague have suggested that P_i_ binding precedes ADP binding during ATP synthesis [31] and that *two* catalytic sites may carry out the ATP synthesis reaction *simultaneously* [3]; in contrast, according to the torsional mechanism, ADP binding precedes P_i_ binding during physiological ATP synthesis, and ATP is synthesized during the loose to tight *conformational transition* of a β catalytic site and only one of the three catalytic sites (the T-site), can contain the synthesized, tightly bound MgATP at one time [1, 2, 17]. From the unified theory above, after incorporating the C′ state, predictions 8 and 9 [1] yield the following sequence of conformations that a single catalytic site cycles through during physiological ATP synthesis:

(3) $$\beta_{\text{E}}\to \beta_{\text{C}}(\text{MgADP})\to \beta_{{\text{C}}^{\prime }}(\text{MgADP}+{\text{P}}_{\text{i}})\to \beta_{\text{TP}-\text{like}}(\text{MgADP}{\text{.P}}_{\text{i}})\to \;\beta_{\text{DP}-\text{like}}(\text{MgATP})\to \beta_{\text{E}}$$

*i.e.* we have five different conformational states (excluding the transition state) to describe the synthesis mechanism. Or, in words, the sequence of conformations that a particular β-subunit passes through during ATP synthesis is O (open, β_E_) to C (closed, β_C_) to C′ (closed, β_C′_) to L (loose, β_TP-like_) to T (tight, β_DP-like_) and back to O (open, β_E_) (Figure 2). The bound nucleotide occupancies of the catalytic sites during ATP synthesis are: no bound nucleotide in β_E_ (open), MgADP in β_C_ (closed), MgADP + P_i_ in β_C′_(closed), MgADP.P_i_ in β_TP-like_ (loose), and MgATP in β_DP-like_ (tight) (Figure 2). Extending the sequence of events at a single β catalytic site to the ATP synthase enzyme during ATP synthesis as a whole (Figure 7 of Ref. [1] is still very much apposite here), it is predicted that the order of the conformational changes of the catalytic sites is O → C → C′, followed by T → O, followed by L → T, and lastly, C′ → L. Note that due to the presence of torsion in the γ-shaft in accordance with a central tenet of the torsional mechanism of ATP synthesis, there is sufficient time (while the bottom of γ is rotating step-wise in ∼15°/∼30° intervals but the top of γ is stationary) for the formation of the intermediate conformations C and C′ in which MgADP and P_i_ can bind respectively. In the binding change mechanism, on the other hand, the postulate of free rotation of γ does not provide sufficient time for the binding of substrates, and this is another reason why the concept of free rotation, though a part of current scientific dogma, leads to acute mechanistic difficulties.

A major problem with the sequence for synthesis in Eq. (2) [33] is that it is enabling ADP to bind to an open site, P_i_ also to bind to that site, ADP and P_i_ to be activated for nucleophilic attack, transition state formation, and ATP synthesis, all in a single binding change. Since P_i_ binding and subsequent steps require energy, this is very difficult, if not impossible to conceive. The latest version of the binding change mechanism [39] has also incorporated this sequence of conformations, a major departure from its earlier stance of several decades [7], without any justification (except perhaps to bring it in-line with the proposal in Eq. (2) [33], though no such, or any other, explanation was offered [39]), which was criticized as even more problematic for it than earlier versions (see p. 74 of Ref. [1], and further related aspects on pp. 135–136 of Ref. [2]). If, indeed, all the above changes can take place in a single O → T binding change, as hypothesized, then what is the need to have three catalytic sites in the F_1_-portion of the enzyme, and what is the function of the L-site? Thus this basic structural feature of the F_1_-ATPase will then defy explanation. Note that none of these problems beset the torsional mechanism and the unified theory, because each of the above chemical steps takes place in a different conformation ([1, 2] and Eq. (3) of this work and the description below it). A possible reason for the structural interpretations could be that the mitochondrial F_1_ X-ray structures [33, 40] are of an “MgADP-inhibited state” (as mentioned in the original 1994 native mitochondrial F_1_ structure paper [40]), as they were carried out with various inhibitors. I propose that the state captured in the crystal structures mimics a metastable, post-hydrolysis, pre-product release state after ∼80° rotation of the γ-subunit.

In the light of the unified theory and the above discussion, the sub-predictions in prediction numbers 8 and 9 for the hydrolysis mode (p. 80 of Ref. [1]) must be revised. Within the framework of that paper, we can now state that the order of conformations that an F_1_-ATPase catalytic site passes through during steady-state V_max_ hydrolysis is O to T to L to C and back to O. The bound nucleotide occupancies of the catalytic sites during steady-state V_max_ ATP hydrolysis by F_1_-ATPase are: no bound nucleotide in β_E_ (open), MgATP in β_DP-like_ (tight), MgADP.Pi in β_TP_ (loose), and MgATP in β_C_ (closed). Starting with the enzyme in the L, O, T resting state (as on page 79 of Ref. [1] seen from the F_1_ side, except that now γ will rotate clockwise) with L containing bound MgADP, O either containing no bound nucleotide or containing bound MgATP (depending on whether the MgATP bound to O before/during, or after the clockwise ∼80–90° γ rotation sub-step, but since the ɛ-subunit is still interacting with this catalytic site (site 3), it is called O here), and T (*i.e.*, site 1 which is β_DP-like_) containing bound MgATP. After ADP-ATP exchange has occurred in the L-site (Section 2.4.3), it contains bound MgATP. The top of γ (γ_t_) rotates ∼80–90° clockwise due to ADP-ATP nucleotide exchange occurring in the L-site. Upon γ_t_ movement clockwise, the L-site changes to the C conformation. The ɛ-subunit and the bottom of γ (γ_b_) have not rotated yet, and the ɛ-subunit continues to interact with O (β_E_). After the first rotation sub-step, with γ paused at an angular position of ∼80–90°, T changes to the L conformation and ATP hydrolysis occurs during the catalytic dwell, and now the new L-site contains bound MgADP.P_i_. This is the snapshot (in the overall structural sense, in terms of the angular position of the single-copy subunits γ and ɛ, and conformationally of the β-catalytic sites) of the native mitochondrial F_1_-ATPase structure of 1994 [40] in the so-called “MgADP-inhibited state,” though of course not in terms of nucleotide occupancies. The O-site (corresponding to β_E_ in the structure) is either empty of bound nucleotide (as in the structure) or may contain bound MgATP, the L-site contains bound MgADP.P_i_ (and corresponds to β_TP_ in the structure where it contains a bound MgAMP-PNP analog), and the C-site contains bound MgATP (equivalent to β_DP_ in the structure where, however, it is occupied by bound MgADP). It should be carefully noted that this catalytic site, though structurally tight compared to the other two catalytic sites in the structure, is *not the T-site (site 1)*. After release of ∼9 kJ/mol of MgATP hydrolysis energy has helped to reduce P_i_ binding in the L-site and the other ∼9 kJ/mol has been funneled via ɛ-β_TP_ interactions (through the interaction of the helix tip ending in Met-138 of the ɛ helix-turn-helix motif) to the O-site (which, together with the binding energy of MgATP binding in the site, helps to close it and break the ɛ-β_E_ interaction occurring via ɛ-Ser-108), the ɛ and γ_b_ move away clockwise. Thus, now O has changed its conformation to T, and contains tightly bound MgATP. Meanwhile, ɛ and γ_b_ continue their clockwise rotation to an angular position of ∼80–90°. *Concomitantly*, the phosphate is released from the L-site (which now contains bound MgADP) and the ∼18 kJ/mol is used to rotate γ (both γ_t_ and γ_b_) and ɛ clockwise from ∼80/90° to 120°. The interaction of ɛ with the C-site changes it to an O-site, from which the bound MgATP is released, and thus the O-site is empty of bound nucleotide, and the resting/ground state of the enzyme from which we initiated the cycle has now been regained, but with a 120° clockwise shift (looking from the F_1_-side), and the new L-site is ready to release its ADP at the 120° angular position of γ, participate in the next round of ADP-ATP exchange, and thus the next one-third part of the hydrolysis catalytic cycle can begin afresh. A similar mechanism operates in F_1_F_O_ [11] except that, after phosphate release from the L-site, γ_t_ rotates clockwise from ∼80/90° to 120° while ɛ and γ_b_ rotate clockwise in ∼15/∼30° steps from 0° until they reach 120°.

It should be noted that if there are high (∼mM) concentrations of Mg^2+^ and ATP in the surrounding medium, then a favorable concentration gradient for release of the MgATP from the O-site into the medium may not exist, in which case the O-site will contain MgATP in it, and the mechanism will be tri-site. In any case, to obtain *steady-state hydrolysis activity*, *i.e.* if the rotation of γ and ɛ is to occur continuously over several cycles, and not stop after ∼80°, then the O-site will have to be filled by medium MgATP, and the steady-state hydrolysis mechanism by F_1_-ATPase will have to be tri-site. This is because if the O-site is empty, then upon the O → T transition of the catalytic site (*i.e.*, without requiring MgATP binding to the O-site if such a transition can occur at all, which itself is very unlikely in the first place), the T-site will be empty (instead of containing tightly bound MgATP), and hence no hydrolysis and therefore no phosphate release can take place after the T → L transition of the catalytic site upon ∼80° rotation of the γ-subunit driven by ADP-ATP exchange in the L-site of F_1_-ATPase. Hence, in either case, the ∼40° rotation of γ will not take place, and the F_1_ enzyme molecule will be trapped at an angular position of γ of ∼80°, and no steady-state hydrolysis activity will be observed. Hence the criterion of observing steady-state rotation and obtaining steady-state ATPase activity in F_1_-ATPase imposes the requirement that all three catalytic sites be filled with bound Mg-nucleotide, as discussed above. In other words, the mechanism of steady-state hydrolysis by F_1_-ATPase has to be tri-site, as found experimentally [3, 32]. However, in such a tri-site hydrolysis mechanism by F_1_-ATPase, the MgATP bound to the O-site will be released as MgADP from the L-site after ∼240° clockwise rotation of the γ-subunit (looking from the F_1_ side) and not wait another ∼120° (*i.e.* till ∼360°) for release until the L → C → O transition has occurred (or be released as MgADP between 320–360°, as suggested [35]), because of the operation of the *special phenomenon of ADP-ATP nucleotide exchange in the L-site (site 2) in the hydrolysis mode*. Thus, at high (∼mM) concentrations of Mg^2+^ and ATP in the medium, there exists a favorable concentration gradient for bound ADP in the catalytic site to be released into the medium and a favorable concentration gradient for entry and binding of ATP from the medium into the catalytic site. It should be noted that MgATP bound in catalytic sites of the enzyme will have very little tendency to be released into the medium because the concentration gradient favors the binding of MgATP into the catalytic sites rather than its release from the sites. Thus, the ATP which exchanged into the L-site for ADP may remain bound as MgATP or be released after 120° rotation of γ and ɛ, when the catalytic site changes conformation from L to C and then to an O-site upon interaction with the ɛ-subunit, depending on the *local* concentration gradient seen by the bound MgATP. If it is released, it will have to rebind in order to continue steady-state hydrolysis. However, as discussed above, at high medium Mg^2+^ and ATP concentrations of >1 mM that lead to maximal hydrolysis rates by F_1_-ATPase, release of bound MgATP from a catalytic site will be prohibited due to the presence of an adverse concentration gradient of Mg^2+^ and ATP. On the other hand, unbinding and release of ADP from a catalytic site and entry and binding of ATP into an unoccupied catalytic site will be strongly favored due to the presence of a downhill concentration gradient of ADP from the site to the medium and likewise a downhill concentration gradient of ATP from the medium to the site. Looked at in another way, exchange of bound ATP with medium ATP is not a real exchange; hence during hydrolysis under conditions of high medium ATP, nucleotide exchange is meaningful only from a catalytic site containing bound ADP, *i.e.* site 2 in F_1_-ATPase.

In the above mechanism for hydrolysis of ATP by F_1_ or F_1_F_O_, if a second, different definition of n-site is used, then the first *initiation* step of ∼80° rotation of γ may be termed tri-site or bi-site respectively, depending on whether MgATP binds to site 3 (O-site) *before* the ∼80° rotation sub-step due to ADP-ATP exchange in site 2 (L-site), or *after* the ∼80° sub-step and before the ∼40° sub-step due to the ATP hydrolysis energy released upon phosphate release in site 2 (L). The second sub-step of ∼40° rotation occurs under tri-site conditions, because the second ∼40° γ-ɛ sub-step has to wait for MgATP binding in the third unoccupied site (O), and until this happens, the enzyme will be trapped in the so-called “MgADP-inhibited state.” Besides, the open hinged-out O-site will offer steric hindrance to *continual* γ rotation, which cannot be relieved without MgATP binding to the O-site. Thus*, leaving the O-site unfilled will not lead to steady-state turnover*, and the rotation and hydrolysis will cease very soon, for example after ∼80° rotation of the top of γ. Moreover, at high millimolar concentrations of Mg^2+^ and ATP in the medium, when there is more than sufficient MgATP present to fill all three catalytic sites, there is no reason why the MgATP will wait to bind to site 3 rather than taking the first available opportunity to bind to it (for example before the beginning of the ∼80° sub-step of rotation itself). Finally, since, due to the absence of a driving force for release, there is no reason why MgATP bound in the O-site should unbind and be released when the medium itself contains high Mg^2+^ and ATP concentrations, it logically implies that MgATP binding to site 3 (O-site) does not occur every 120°, and therefore this substrate binding event to site 3 cannot be the energy source that causes complete or partial rotation of γ in each 120° cycle. In any case, an appropriate definition of n-site is the *maximum* number of β catalytic sites of the F_1_/F_1_F_O_ enzyme that are required to be filled with Mg-nucleotide in order to observe continuous steady-state γ or γ-ɛ rotation and/or obtain steady-state ATP hydrolysis rates. This is also consistent with the definition espoused earlier [p. 135 of Ref. 2]. Note also that the ADP itself cannot unbind and exit from site 2 until after the ∼40° rotation sub-step has occurred: during the ∼40° sub-step of rotation, between the angular positions of γ from ∼80–90° to 120°, the binding of MgADP progressively starts loosening in its site, and it can exit from L at an angular γ position of 120°. Thus the mechanism of hydrolysis in a steady-state mode by F_1_ or F_1_F_O_ is tri-site, and the fraction of the total enzyme molecules that will incur three-site filling (given the known nucleotide binding affinity (K_d_) properties of the three F_1_ catalytic sites) and therefore exhibit steady rotation in a rotation assay and steady-state V_max_ hydrolysis activity will depend on the experimental conditions (e.g. the Mg^2+^ and ATP concentrations) in the medium.

Finally, it should be noted that the hydrolysis mechanism postulated here is microscopically the reverse of the ATP synthesis mechanism postulated by the torsional mechanism of energy transduction and ATP synthesis, as can be clearly seen by carrying out the cycle of Figure 7 on page 79 of Ref. [1] in the reverse clockwise direction as per the torsional mechanism of ATP hydrolysis presented here. The fact that microscopic reversibility is satisfied by the molecular mechanisms of the synthesis mode and the hydrolysis mode, each of which was derived independently and at different times, is another important point lending great confidence to the mechanistic correctness of the proposals of the torsional mechanism and the unified theory.

The hydrolysis mechanism above is different from and simpler than that proposed by Senior and colleagues (reviewed in Ref. [3]), as seen for example from the number of steps required for the interconversion of sites during V_max_ hydrolysis in the two mechanisms. A further difference is that in our mechanism, as in our earlier scheme [1, 2], two ATP molecules and one ADP molecule are bound in time average at the catalytic sites during steady-state hydrolysis, unlike two ADP and one ATP in Senior’s mechanism [3]. Moreover, the hydrolysis bond cleavage step occurs *before* the completion of the ∼80° sub-step of γ rotation in Senior’s model [3] as well as in the Walker-Leslie model [33] and both ATP binding to the lowest affinity site and ATP hydrolysis at the highest affinity site acting in sequence drive the ∼80° sub-step of γ rotation, and in fact, the hydrolysis step is held directly responsible for the major fraction of the 120° γ rotation in these models [3, 33]. However, in the torsional mechanism of ATP hydrolysis and the unified theory, the chemical hydrolysis step occurs during the catalytic dwell and *follows* the completion of the ∼80° sub-step of γ rotation and hence is not linked to the ∼80° sub-step of γ rotation, in agreement with single molecule experiments [30]. It should also be pointed out that the ATP hydrolysis mechanism postulated by Menz *et al.* [33] incorporates five different conformational states, while only *four* different conformations are required in our torsional mechanism of ATP hydrolysis presented in this section, and hence our molecular mechanism offers the *minimum* number of different conformational states required for steady-state ATP hydrolysis, because three conformational states (O, L and T), as envisaged by the binding change mechanism [7], are not sufficient, as discussed earlier in some detail [1, 2, 16–19]. Moreover, the mechanistic scheme for ATP hydrolysis given in Menz *et al.* [33] has additional states such as L′ and T* which have not been observed by the leading structural group in the field despite the passage of almost a decade, and hence, for all the reasons given above, is neither the simplest nor the shortest pathway that explains the observations. It is not clear why a longer pathway is being proposed, rather than the simpler and shorter pathway offered by the torsional mechanism. In fact, the pathway postulated by the torsional mechanism is consistent with all the structural observations. Further, the mechanism in Figure 4 of Ref. [33] can be reduced to a bi-site mechanism. (Note that the L-site simply contains ATP in all the four diagrams of Figure 4 [33]). Thus, the mechanism appears tri-site only because of the labeling. It has no real useful information above a bi-site mechanism. The objections of Berden [41, 42] to certain mechanistic schemes may also be pertinent in this context. On the other hand, the torsional mechanism is a true tri-site mechanism, as repeatedly emphasized [1, 2, 16–18], and also explains why the ATP synthesis mechanism should be tri-site.

### 2.6. Kinetic Analysis of ATP Hydrolysis by F_1_-ATPase

**Figure 3.**
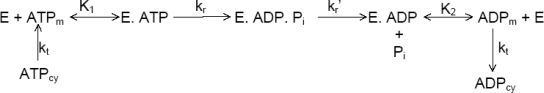
Kinetic Scheme for ATP Hydrolysis by F_1_-ATPase

A kinetic scheme based on a general sequence of events leading to ATP hydrolysis which considers irreversibility of the catalysis steps (Section 2.5.2), as proposed earlier by us [1, 13, 15, 16] was developed, as depicted in Figure 3. In this kinetic scheme, E represents the F_1_-ATPase enzyme molecule, e.g. after MgADP has been released from the L-site, E.ATP the enzyme-ATP complex, e.g. containing MgATP bound in the T-site after the O → T conformational transition of the catalytic site, E.ADP.P_i_ the enzyme-ADP-inorganic phosphate complex, e.g. the catalytic site occupancy in the L-site after ATP hydrolysis has occurred during the catalytic dwell, and E.ADP the enzyme-ADP complex, e.g. after phosphate release from the L-site during the P_i_-release waiting dwell (Sections 2.4.3 and 2.5.2). K_1_ and K_2_ denote the dissociation constants of the corresponding elementary steps (Figure 3). k_r_ denotes the rate constant for conversion of E.ATP to E.ADP.P_i_, and k_r_′ the rate constant for conversion of E.ADP.P_i_ to E.ADP. (It should of course be understood that in the kinetic scheme of Figure 3, the shorter symbols of ATP, ADP.P_i_ and ADP stand for the MgATP, MgADP.P_i_ and MgADP, as the true substrate of the enzyme is the Mg-nucleotide). k_t_ stands for the constant of proportionality relating the rate of *transport* of adenine nucleotides by the adenine nucleotide transporter (ANT) for the case in which the operation of the ATP synthase has been reversed *in vivo* (e.g. in necrosis and other diseases) to the corresponding adenine nucleotide concentration gradients (Figure 3). The subscript cy represents the concentration in the cytoplasm of the cell and the subscript m denotes the concentration in the organelle (e.g. mitochondrion) or the medium *in vitro* in which the hydrolysis reaction takes place.

Now a mathematical analysis of the kinetic scheme of Figure 3 will be carried out. For steady-state operation, the rate of ATP hydrolysis and rates of transport, binding and dissociation of ATP and ADP are equal. Thus v_hyd_, the rate of ATP hydrolysis can be written as

(4) $$\nu hyd=k_{t}(ATP_{cy}-ATP_{m})$$

(5) $$\nu hyd=k_{r}E.ATP$$

(6) νhyd=kr'E.ADP.Pi

(7) $$\nu hyd=k_{t}(ADP_{m}-ADP_{cy})$$

If E_0_ represents the total enzyme concentration, then, from a material balance on E, we have

(8) $$E_{0}=E+E\cdot ATP+E.ADP.P_{i}+E.ADP$$

*i.e.*,

(9) E0=E+E(ATPm)K1+[E(ATPm)K1](krkr')+E(ADPm)K2

Therefore,

(10) E=E0[1+ATPm(1+krkr')K1+ADPmK2]

Combining Eqs. (5) and (10), we have

(11) νhyd=krE0ATPm[K1+ATPm(1+krkr')+ADPm(K1K2)]

Eq. (11) can also be re-written as

(12) νhyd=krE01+krkr'ATPmATPm+K11+krkr'+ADPmK1K2(1+krkr')

For *in vitro* hydrolysis, Eq. (11) or Eq. (12) is sufficient, but for the general case in the presence of transport steps, we can write ATP and ADP concentrations in the organelle in terms of the ATP and ADP concentrations in the cytoplasm of the cell. This results in

(13) νhyd=krE0(ATPcy−νhydkt)[K1+ATPcy(1+krkr')+ADPcy(K1K2)+νhydkt(K1K2−1−krkr')]

For the case of fast diffusion/exchange of adenine nucleotides into and out of the F_1_-ATPase (e.g. by the action of the adenine nucleotide transporter in mitochondria), *i.e.* for large k_t_, we finally obtain

(14) νhyd=krE01+krkr'ATPcy[ATPcy+K1+krE0kt1+krkr'+ADPcyK1K2(1+krkr')]

or

(15) $$\nu hyd=\frac{V_{\text{max}}ATP_{cy}}{\left[ATP_{cy}+K_{M}(1+\frac{ADP_{cy}}{K_{I}})\right]}$$

with the enzymological parameters

(16) Vmax=krE01+krkr'

(17) KM=K1+krE0kt1+krkr'

(18) $$K_{I}=K_{2}+\frac{k_{r}E_{0}}{k_{t}}\frac{K_{2}}{K_{1}}$$

Eq. (14), or equivalently Eq. (15) – Eq. (18) constitute the final result of the mathematical analysis of the kinetic scheme for the general case (with transport steps). For the *in vitro* case, Eq. (12) is itself the principal result, where the subscript m refers to the ATP and ADP concentrations in the medium. Comparing Eq. (12) with an equation similar to Eq. (15) in which the subscript cy is replaced by the subscript m yields the enzymological parameters for the *in vitro* case, *i.e.*

(19) Vmax,in−vitro=krE01+krkr'

(20) KM,in−vitro=K11+krkr'

(21) $$K_{I,in-vitro}=K_{2}$$

The *in vitro* result (Eqs. (19)–(21)) is a special case of the general result in the presence of transport steps, as can be readily seen by putting k_t_ very large (*i.e.* k_t_ → ) in Eqs. (17) and (18). Thus the general result reduces to the *in vitro* result when there are no transport barriers and ATP_cy_ = ATP_m_ and ADP_cy_ = ADP_m_, as it should, based on physical intuition. Thus, the principal results of our model show hyperbolic Michaelis-Menten kinetics with respect to ATP in the hydrolysis mode at different ADP concentrations in the medium and show the occurrence of competitive inhibition of F_1_-ATPase by MgADP as the inhibitor in the steady-state hydrolysis mode. These results are consistent with the known experimental fact that MgADP is a competitive inhibitor of ATP hydrolysis activity. This implies that the MgADP competes with the substrate MgATP, or the bound MgADP changes the conformation of the site meant for substrate MgATP binding, thereby not allowing the MgATP to bind to the site. Hence, unless product MgADP is released from the catalytic site, binding of substrate MgATP cannot occur. Thus, an important consequence of the competitive inhibition is the order imposed on binding and release events during steady-state hydrolysis, *i.e.*, product MgADP release must precede substrate MgATP binding. As the analysis is based on a general kinetic scheme that is applicable to all mechanisms, the above implication is valid irrespective of the specific mechanism.

As discussed above, the above mathematical analysis of the general kinetic scheme provides deep insights into the sequence of events in steady-state V_max_ ATP hydrolysis and has important biological implications for hydrolysis mechanisms because it shows that product release must precede substrate binding. This contradicts all current mechanisms and models (except Nath’s mechanisms) because in all of these, substrate binding is postulated to precede product release or be simultaneous with it during steady-state hydrolysis. This also suggests that if, in certain instances, e.g. at high medium Mg^2+^ and ATP concentrations, MgATP binding to the O-site along its concentration gradient (from medium to site) were to precede MgADP release down its own concentration gradient (site to medium) from the L-site (this could happen since the events of MgADP release from L and MgATP binding to O occur due to their own, *independent* driving forces, as discussed in Section 2.5.2, and one does not influence the other) it will not violate the principal result of the kinetic model of this section if substrate binding to the O-site is not the event that donates energy and helps cause the partial or complete ∼80° rotation of the γ-subunit and catalysis by F_1_-ATPase. However, if MgADP release from L precedes MgATP binding to O in F_1_-ATPase, then it is far more likely that MgATP binds to L before it binds to O, because of the much higher nucleotide binding affinity of the L-site over the O-site. (Otherwise, the mechanism would suffer from the same flaws pointed out by us earlier in relation to the binding change mechanism [1], for example of relying on unlikely and improbable events such as filling the lowest-affinity site 3, yet keeping the higher-affinity site 2 empty of bound nucleotide). Hence our mechanistic interpretation of the above is that MgATP needs to bind to site 3, and thereafter an MgATP molecule has to either stay bound in site 3 after the L to C to O conformational transition of a catalytic site, or if it is released from site 3 after the L to C to O conformational change of the catalytic site during activity, it has to rebind to site 3, in order to ensure steady-state hydrolysis, as discussed in detail in Section 2.5.2. However, whether MgATP binds to site 3 before, after, or simultaneously with MgADP release from site 2 is immaterial to the process, because substrate binding to site 3 is not the event that donates energy to cause rotation of the γ-subunit and hence the MgATP that binds to site 3 is not the true substrate molecule (*i.e.* the E.ATP in the kinetic equations, e.g. in Eq. (5) and Eq. (8) of this section) that causes rotation and catalysis in F_1_-ATPase. In fact, in the absence of a driving force for release of bound MgATP in site 3 into a medium itself containing high Mg^2+^ and high ATP concentrations, MgATP binding to site 3 is not expected to occur every 120° and hence for all the reasons given above, it cannot be the binding event that causes complete or partial γ rotation in each 120° cycle during ATP hydrolysis. On the other hand, MgADP release from the L-site and subsequent MgATP binding to the L-side, *i.e.* the process of ADP-ATP nucleotide exchange in site 2 has sufficient energy to rotate the γ-subunit by ∼80° and is also in agreement with the order of binding and release events imposed on all hydrolysis mechanisms by kinetic analysis.

## 3. Consistency of Current Mechanisms of ATP Synthesis and Muscle Contraction with Key Experimental Data

### 3.1. Bioenergetics

#### 3.1.1. Nucleotide Binding Affinity (K_d_) Measurements at the Catalytic Sites of F_1_-ATPase

As discussed in Sections 2.3 and 2.5.2, the binding change mechanism was based on the tenet that the ATP synthase can stabilize the MgATP differentially with respect to MgADP and P_i_ by binding the MgATP very tightly (e.g. in a highly sequestered site where the reaction is at equilibrium with K_eq_∼1, and there is free dynamic reversal on the enzyme surface), and that energy input occurred not at the bond formation step during ATP synthesis but at the step of product MgATP release [7]. This can be verified by evaluating measurements of nucleotide binding affinity to the catalytic sites of F_1_-ATPase. Earlier measurements of the binding affinity of MgATP to site 1 in mitochondrial F_1_ indicated a K_d1_ value of ≤ 1 pM [45]. Since MgADP was taken to bind with a K_d2_ of ∼10 μM in site 2, a difference of binding energy ≥ RT ln (K_d2_ / K_d1_), *i.e.*, ∼35 kJ/mol was inferred. However, as reviewed by us previously [1, 2], very careful measurements of binding affinities over a decade of experimentation through the development of a true equilibrium technique using optical probes that could directly monitor catalytic site occupancy, Senior and colleagues have proved that the MgATP binds to site 1 of *E. coli* F_1_ with a K_d_ of only 0.02 μM (and not 1 pM), and to site 2 with a K_d_ of ∼1 μM, and that MgADP binds to site 2 with a K_d_ of ∼25 μM [1–3]. Assuming additivity of binding energies in site 1 and site 2, calculation reveals that the binding energy of phosphate in its site is ∼7–10 kJ/mol, and that the binding energy difference between MgATP in site 1 and MgADP.P_i_ in site 2 is only ∼7–10 kJ/mol (and not the ≥ 35 kJ/mol postulated in the binding change mechanism). Thus this postulate of the binding change mechanism is not consistent with the biochemical nucleotide binding affinity (K_d_) experimental measurements, which are now generally accepted. It should be noted that there is no difficulty for the torsional mechanism, which is consistent with these experiments. The sequential ∼9 kJ/mol + ∼9 kJ/mol released upon ATP hydrolysis (Section 2.4.3) are immediately used to weaken phosphate binding and weaken a subunit-subunit interaction and lead to no net free energy changes, while the ∼18 kJ/mol of the hydrolysis free energy is kept stored as potential energy and is released at a later step upon phosphate release, causing rotation of a subunit, or is stored in a localized region of the macromolecule for performance of useful work subsequently upon release of the stored energy, as discussed in Sections 2.4.2, 2.4.3, 2.5.1 and 2.5.2, again leading to a net change of zero free energy. The concomitant increase of binding energy of MgATP in site 1 by ∼9 kJ/mol (over MgADP.P_i_ in site 2) during physiological ATP synthesis during the L to T transition (Section 2.4.1), and the concomitant reduction of MgADP binding in site 2 by ∼9 kJ/mol during the bond cleavage step in hydrolysis during the T to L transition (Section 2.4.3) is consistent with the nucleotide binding affinity measurements on F_1_-ATPase, and the binding energy difference of ∼7–10 kJ/mol above.

Finally, it should also be pointed out that the postulate of a highly sequestered site binding MgATP (or an ATP analog) with a K_d_ of 1 pM or lower is not consistent with recent experimental data which show that the K_d_ for MgITP binding in the catalytic site with the highest affinity of *E. coli* F_1_ is only 0.33 μM, yet MgITP is readily synthesized by *E. coli* ATP synthase [3]. In summary, it may be stated that the binding change mechanism is not consistent with basic biochemical data on nucleotide binding affinities at the catalytic sites of F_1_-ATPase and hence, in addition to the plethora of reasons given in this paper and in earlier papers [1, 2, 10, 11, 13, 16–18], it is absolutely clear that the mechanism requires revision.

#### 3.1.2. Structure of Mitochondrial F_1_-ATPase with Nucleotide Bound to All Three Catalytic Sites

It has already been reviewed how the F_1_-ATPase structure containing nucleotide bound to all three catalytic sites is consistent with the torsional mechanism, but completely inconsistent with the binding change mechanism (p. 73 of Ref.[1]). It should also be mentioned that upon superposition of the more recent structure above with the original native mitochondrial F_1_ structure, it was stated in 2001 (p. 338 of Ref. [33]) that (in the two structures) “the two conformations of the γ-subunit are related by a rotation that varies in magnitude along the length of the C-terminal helix, ranging from less than 1o for the final residues (γ 259–272) to a maximum of about 20° for residues γ 234–244 (which form the coiled coil with residues γ 20–10).” Further, it was stated that “the variation in the rotation angle implies that the coiled-coil is slightly more twisted in the (ADP.AlF_4_^−^)_2_F_1_ structure than in the native form,” and further on that “the rotation is not uniform over the length of the γ-subunit.” These were important observations being made for the first time, thanks to the possibility of comparison afforded between two γ conformations due to the solution of the second key structure. However, all the longer statements within quotes could be considerably condensed by calling it “torsional strain” in the γsubunit, as predicted by the torsional mechanism from inception. This central characteristic led to the title of one of our earlier papers [18] and the naming of the mechanism as “the torsional mechanism” from 1999, and subsequently as “the torsional mechanism of energy transduction and ATP synthesis”. In fact, the experimental observations made for the first time in 2001 [33], and inserted within quotes here, define, (and are the very inherent attributes of) torsion, and thus offer further important evidence in support of the torsional mechanism. It should however be added that, in contrast, the rotation of the γ-subunit upon “substrate binding” (or rather, ADP-ATP exchange), and “product release” (*i.e.*, P_i_ release) steps is not “small,” as postulated [33], but as large as ∼80° and ∼40° respectively, as evident from the unified quantum theory of protein molecular machines synthesizing/hydrolyzing ATP developed in this paper. In summary, the torsional mechanism is in agreement with all the structural observations. We believe that interpretation of structural, biochemical and biophysical data in the light of the torsional mechanism (as opposed to the binding change mechanism) will help solve the longstanding difficulties in the field and also lead to considerable and rapid progress in the near future.

### 3.2. Muscle Contraction

#### 3.2.1. Contraction Characteristics and ATPase Activity of Muscle Fibers in the Presence of Antibody to Myosin S-2

In an interesting experiment, Sugi, Harrington, and colleagues examined the contraction characteristics and MgATPase activity of glycerinated muscle fibers prepared from rabbit psoas in the presence and absence of polyclonal antibody directed against the S-2 region of myosin [44]. Their results indicated that Ca^2+^-activated force and muscle fiber stiffness were uncoupled from MgATPase activity in the antibody-treated muscle fibers. In other words, when anti-S-2 antibody attaches to the S-2 coiled coil of myosin molecules, their heads still hydrolyze ATP, but no longer contribute to force production and muscle fiber stiffness. This experimental fact cannot be explained by previous mechanisms of muscle contraction, including the lever arm model, which do not postulate any role for the S-2 coiled coil in the generation of elementary contractile force by myosin. The question that arises, which is unanswered by previous mechanisms and biochemical/ mechanochemical schemes, is how antibody attached to the S-2 coiled coil of a myosin molecule could exert a long-range effect on the mechanochemical properties of the myosin heads? This question is readily answered by the rotation-uncoiling-tilt (RUT) energy storage mechanism of muscle contraction, in which a transmission of the free energy release of MgATP binding, hydrolysis and phosphate release by the physicochemical processes occurring in the myosin head takes place all the way to S-2 via the helical structure of myosin and the S-1–S-2 hinge (*i.e.* head-rod junction), stores the free energy in a high-energy uncoiled state of the S-2 coiled coil, which is then released back from S-2 to S-1 and the actomyosin contacts via the S-1–S-2 hinge after the myosin heads have bound tightly to actin sites, and causes a powerstroke of the myosin heads bound to actin with the S-1–S-2 hinge as fulcrum. Thus, the RUT energy storage mechanism of muscle contraction can explain all the experimental facts usually given in support of the lever arm model, and also other longstanding experimental facts that have proved very hard to explain by other mechanisms of muscle contraction. Sugi and Harrington [44] concluded that the myosin hinge plays an essential role in muscle contraction, and that much more attention must be paid to the function of this region. To this we would like to add that the S-2 coiled coil also plays an essential role during muscle contraction by storing the free energy of the MgATPase processes occurring in the myosin head, and recommend that the S-2 region also needs to be paid greater attention by the muscle contraction and motility community, and that the RUT energy storage mechanism of muscle contraction can serve as a basis for further experimentation in this field.

#### 3.2.2. Single Molecule Imaging

Several single-molecule imaging studies in the field of motility have been carried out in the last ten years, though most of them have employed unconventional myosins (such as myosin V and myosin VI), or kinesins, or other processive molecular motors as model systems (Section 6), partly because of their larger step size (often tens of nanometers), compared to myosin II, and their processive character. A powerful single-molecule study that deals with muscle myosin as the experimental system is that by Yanagida and his coworkers [45], where the chemical ATPase and mechanical events were simultaneously measured in single myosin molecules during force generation and interaction with actin. The experiment reveals unambiguously that myosin II can produce force several hundred milliseconds *after* MgATP hydrolysis and release of bound nucleotides. This important finding [45] does not support several existing models in which force production is directly coupled to the release of bound ligands. The delay in force generation after release of bound ligands from the catalytic site in myosin S-1 points to the ability of myosin II to store chemical energy from ATP binding, hydrolysis and product release and subsequently perform useful work with the stored energy, as postulated in a central tenet of the RUT energy storage mechanism of muscle contraction [2, 10, 11, 14].

## 4. Fundamental Consequences of the Torsional Mechanism, the Rotation-Uncoiling-Tilt Energy Storage Mechanism, and the Unified Theory for Molecular Mechanisms of ATP Synthesis/Hydrolysis and Muscle Contraction

As discussed in Sections 2.1, 2.3, 2.4.2 and 3.2.2, previous mechanisms of ATP synthesis and muscle contraction have not felt the necessity to include definite aspects of energy storage within the enzyme molecule, which is of central importance in the torsional mechanism of energy transduction and ATP synthesis, the rotation-uncoiling-tilt energy storage mechanism of muscle contraction, and the unified theory. This has fundamental consequences [10] because previous mechanisms can be shown to violate the first and second laws of thermodynamics.

### 4.1. Violations of the First Law of Thermodynamics by Previous Mechanisms of ATP Synthesis and Muscle Contraction

In the binding change mechanism of ATP synthesis, the γ-subunit rotates freely (“spins like a top” in one of the descriptions). However, it is not possible to transduce the rotational kinetic energy of the spinning shaft into the stored internal energy/potential energy of ATP, as discussed in Section 2.3. In fact, as the shaft slows down (as it must, every 120°, since the rotation is in discrete steps), the rotational kinetic energy will be thermalized and dissipated as heat, and not converted to useful work or transduced to another form of stored energy. We are then left with binding energy alone as the sole source of useful external work. But calculation based on experimental nucleotide binding affinity values (Section 3.1.1) and use of the expression ΔG = RT ln (K_d2_ / K_d1_) shows clearly that the binding free energy difference between loose and tight catalytic sites in the F_1_ portion of ATP synthase is only ∼7–10 kJ/mol and is thus *grossly insufficient* to account for the required stabilization of ∼55 kJ/mol postulated in the previous mechanism, or, for that matter, of even the standard state value of ∼35–36 kJ/mol in the synthesis mode. Further, additional energy is required to weaken subunit-catalytic site (e.g. ɛ-β) interactions, and to bind inorganic phosphate to the F_1_ catalytic site in the complete enzymatic cycle. Thus, the binding energy of MgADP/MgATP to the catalytic sites is grossly insufficient to make ATP and unless another source of energy is added, the overall energy balance is not satisfied, and therefore the first law of thermodynamics is violated. This violation of the law is removed if internal energy, specifically as torsional energy stored in the γ-subunit of F_1_F_O_-ATP synthase as in the torsional mechanism, is added to the MgADP binding energy. However, it will not be sufficient for previous mechanisms to add an energy source in a vague way to satisfy the first law of thermodynamics (e.g. “protonmotive force,” which has already been used for free rotation of γ); moreover, to be complete theories, it will be necessary to specify how the energy from a particular energy source is transduced, how and where it is accumulated and stored, what is the mode of transmission of that stored energy to the catalytic sites, how that energy is utilized, and above all, what is the mechanism by which all these events occur? These are basically the very details that have been addressed within the grand framework of the torsional mechanism of energy transduction and ATP synthesis. Thus, previous theories have not synthesized ATP but have only made ADP/ADP + P_i_, but since these claim to have made ATP, they could only have done so by violating the first law of thermodynamics.

The above paragraph implies the binding change mechanism of ATP synthesis constitutes a perpetual motion machine of the first kind. It should be clearly recognized that these are fundamental difficulties with the concepts of previous theories of the ATP mechanism, and are not related to the way in which the ATP synthase enzyme actually works. We should clearly distinguish between these two. In any case, the previous mechanisms are incomplete, have not been cast in molecular terms, are not sufficiently detailed to permit a proper evaluation, and the process of energy transduction in these mechanisms remains a black box. In contrast, the new paradigms of the torsional mechanism and the unified theory open this black box of energy transduction and convert it into a white box, and, without exception, none of these inconsistencies and violations of the universal laws of nature arise [10].

Similar difficulties arise with previous models of muscle contraction, such as the lever arm model. If the energy quanta released at various steps of the ATP cycle are not conserved and stored in the myosin molecule, then the energy whose release would have produced the contractile force and lifted a load a certain distance will be dissipated, and will be unavailable for performing useful external work. Yet, in these models, muscle is supposed to have contracted, lifted a load a certain distance, and done useful work, even though the energy of ATP has been wasted and was not available for performance of that work, in violation of the first law of thermodynamics.

### 4.2. Violations of the Second Law of Thermodynamics by Previous Mechanisms of ATP Synthesis and Muscle Contraction

Fundamentally, as analyzed in Section 4.1, if the energy of the ion gradients or that of ATP binding and hydrolysis is not conserved and stored within the enzyme, then that energy must be dissipated as heat and was not used to perform useful mechanical work. We are then left with thermal energy only as the source of the useful work. Thus, all previous mechanisms of ATP synthesis and muscle contraction (other than Nath’s mechanisms) imply that the electrochemical gradients of ions or the energy of ATP is first converted into thermal motions, and only subsequently is it used to carry out useful work [10]. This would violate the second law of thermodynamics since all known biological machines are isothermal. This violation of the second law occurs because it would mean that useful work has been done cyclically using heat energy under isothermal conditions. This is forbidden by the second law of thermodynamics because, according to the law, heat cannot be an energy source unless it flows between a source and a sink at different temperatures. To date, nobody has conceived of thermal gradients in such a small system (a nanomachine enzyme construction), because such gradients, even if they could be produced in the first place, would even out extremely fast, and moreover, it is very difficult to conceive how such thermal gradients in such a small system could ever be directed to the right places/contacts/interaction points to perform useful work, because fundamentally, at the molecular level, heat energy and Brownian motion are random in character. In any case, we aver that biological energy-transducing machines are *not heat engines*. The above fundamental difficulties do not arise in Nath’s mechanisms because there the intermediates are electrostatic/mechanical, and not thermal, and sufficient torsional energy (∼54 kJ/mol in the entire process) is stored in γ-subunit, energetically competent to make the ATP, or sufficient free energy (∼36 kJ/mol) is stored in an uncoiled high-energy state of the S-2 coiled coil in myosin, energetically competent to cause a powerstroke by each head of a double-headed molecule of muscle myosin on actin.

We now see that for a single molecule, it is impossible, according to the second law, to convert energy that has spread over the thermal (rotational, vibrational and translational) degrees of freedom and equilibrated with the surroundings and reached a Boltzmann distribution very fast (in a time t < τ) to a longer-living form of stored energy in a molecular device that lasts or stays stored in the device for a time t > τ. What is thus forbidden by the second law of thermodynamics for single molecules is to take a form of stored energy, allow it to spread over the thermal degrees of freedom, and then try to transduce this thermalized energy, Q, under isothermal conditions, to another form of stored energy, *i.e.*, to an energy that remains stored and lasts longer than the time of thermal exchange. The design principle operative in energy-transducing biological nanomachines, call it the trick of biology, is to *directly transduce stored energy from one form to another*, and not to allow stored energy to spread over and exchange freely with the thermal degrees of freedom of the surrounding bath or reservoir. These fundamental concepts show that biological machines and constructions of molecular dimensions do not work in the same way as heat engines, and in turn enable us to achieve a true understanding of the meaning of the second law of thermodynamics at the molecular level. It shows that it is not sufficient for a fluctuation (e.g. Brownian motion) or other perturbation to lift a microscopic or molecular load; it has to keep the load lifted (or store energy in a device) for a time t > τ, and not lose it to the surroundings as heat within that time. This is because the concepts discussed here and earlier [2, 10, 11] allowed us to make a distinction between heat and work (or stored energy) at the molecular level on the basis of timescales applicable at even a single molecule level. A suitable and concise statement of the second law of thermodynamics applicable to single molecules performing useful work under isothermal conditions then is, “*Single molecules perform useful work by direct transduction of energy from one form to another, and energy once thermalized cannot be transduced into stored energy*.” Previous theories and models of ATP synthesis, muscle contraction, and other related energy transductions, including the binding change mechanism of ATP synthesis and the lever arm model of muscle contraction are Maxwell’s demon machines that violate the second law of thermodynamics because, in all these models, in effect, heat is being converted to work in a cyclic isothermal process. Therefore these theories cannot be correct and need to be replaced by the new paradigm, which will lead to rapid scientific progress in this important interdisciplinary field of science.

### 4.3. Mechanistic H^+^/O, H^+^/ATP and P/O Ratios, Efficiency of Oxidative Phosphorylation, and the Overall Energy Balance of Cellular Bioenergetics

The important question of mechanistic H^+^/O, H^+^/ATP and P/O ratios in oxidative phosphorylation has been the subject of extensive studies for 65 years [1]. To address this aspect, let us consider the dynamically electrogenic but overall electroneutral ion transport proposed within the torsional mechanism of energy transduction and ATP synthesis [1, 2, 13] for the complete process of oxidative phosphorylation *in vivo* in mitochondria. ATP synthesis is regulated by its demand for various cellular processes; when ATP^4−^ is required, it is transported out from the mitochondrial matrix to the cytoplasm along its concentration gradient. The *local* electrical potential thus created drives ADP^3−^ along its concentration gradient to the mitochondrial matrix in exchange for ATP^4−^ by the adenine nucleotide transporter (ANT) [1]. The resulting unbalanced *local* potential is the signal that causes HPO_4_^2−^ to move into the matrix along its concentration gradient, and the OH^−^ produced during ATP synthesis in the F_1_ portion of ATP synthase [1, 17] is driven out of the mitochondria in exchange for the HPO_4_^2−^ by the P_i_/OH^−^ antiporter. The OH^−^ released per ATP produced is neutralized by a proton from the external medium, forming water. Thus, we need ten protons (and not twelve protons as taken earlier [2]) to synthesize three molecules of ATP as clearly seen from the structure of the F_1_-c_10_ complex from yeast mitochondrial F_1_F_O_-ATP synthase [46], *i.e.*, on the average, 3.33 protons to synthesize one molecule of ATP (see Section 2.4.1) and one proton to neutralize the released OH^−^, *i.e.* 4.33 H^+^/ATP *in vitro*. However, in the *in vivo* demand process *utilizing* the ATP transported out of the mitochondrion, the *reverse* process operates, *i.e.* the ATP consumes an OH^−^ and is cleaved into ADP and P_i_. Thus a proton is given back to the external medium (balancing the proton used for neutralization of the OH^−^ in the external medium), and therefore in the *overall* oxidative phosphorylation process *in vivo*, a mean of 3.33 protons is needed to synthesize one molecule of ATP, *i.e.* H^+^/ATP = 3.33, assuming the same c_10_ ring stoichiometry in mammalian ATP synthase as in the ATP synthase from *S. cerevisiae* mitochondria [46]. This then is the value of the mechanistic stoichiometry *in vivo* on the ATPase side. On the redox side, as per the consensus stoichiometry that has evolved after considerable systematic experimental investigation of mechanistic P/O ratios in mitochondrial oxidative phosphorylation [47], ten protons are pumped out by the redox complexes per 2 electrons with NADH-related substrates such as 3-hydroxybutyrate, and six protons are transported from the matrix to the inner membrane against the concentration gradient per 2 electrons with succinate as substrate. Thus the mechanistic H^+^/O stoichiometry *in vivo* is 10 with NADH-related substrates that use site 1, site 2 and site 3 on the redox side, and 6 with succinate that employs only site 2 and site 3. This implies that the mechanistic P/O ratio equals 10/3.33 (= 3.0) and 6/3.33 (= 1.8) for NADH-related substrates and succinate respectively for the *overall* oxidative phosphorylation process *in vivo*. This P/O ratio corresponds to the physiological steady-state mode of operation. The above analysis also carries the important biological implication that the ANT, the P_i_/OH^−^ antiporter and the F_1_F_O_-ATP synthase lie physically close to each other, or in other words form a supercomplex (an ATP synthasome) such that the local potential created by one complex (e.g. ANT) can be sensed by the other complex (e.g. the P_i_/OH^−^ antiporter) and the OH^−^ produced due to the operation of the ATP synthase is immediately funnelled out by the P_i_/OH^−^ antiporter in exchange for the HPO_4_^2−^. This has always been an important prediction inherent in the torsional mechanism [1, 13]. Recently, Pedersen and colleagues have isolated and characterized the mitochondrial ATP synthasome, a 1:1:1 supercomplex of ANT, P_i_/OH^−^ and F_1_F_O_ located near one another in an oblong membrane basepiece, and have provided its first low-resolution (2.3 nm) EM/immuno EM structural model [48]. This important finding of co-localization contradicts the classical view that regards these components as separate entities in the energy-transducing membrane and it is significant that only the torsional mechanism of energy transduction and ATP synthesis is in accord with the new data on membrane association of these entities to form the ATP synthasome supercomplex.

The above has important implications for previous theories of energy coupling, such as the chemiosmotic theory. In chemiosmosis, for each pair of electrons transferred in mitochondrial respiration, up to a maximum of six protons may be produced (H^+^/O = 6) and the number of H^+^ ions transported per O consumed cannot exceed the number of hydrogen carriers present in the respiratory chain. Thus, the number of H^+^ transported per O atom (= 6) includes two transported over NAD, two over flavins and two over quinones and two protons are required for each mole of ATP synthesized from ADP and P_i_ (H^+^/ATP = 2). Several experiments, the energy balance in the torsional mechanism, as well as a nonequilibrium thermodynamic analysis [2, 20, 24, 25] show that these stoichiometries need to be approximately doubled to account for the coupling protons [H^+^/O = 10, H^+^/ATP = 3.33]. These numbers have important thermodynamic consequences because the smaller values of the stoichiometries in chemiosmosis require a larger so-called protonmotive force (in this case by a factor of ∼1.5–2) to make the free energy change energetically competent for ATP synthesis. This poses a formidable problem of energy shortfall for the chemiosmotic theory. The moment experimental evidence and basic nonequilibrium thermodynamic computation that the active proton transport machinery on the redox side must be an ion pump that works with higher stoichiometries than that postulated in chemiosmosis is accepted, the chemiosmotic mechanism of redox loop transport along the respiratory chain breaks down, because there are simply not enough hydrogen carriers to transport 10 protons per oxygen atom. Basically, chemiosmosis does not provide a mechanism to obtain the balance of (10 – 6), *i.e.* 4 protons per O atom. Obtaining these extra protons for coupling presents another insurmountable problem for the chemiosmotic theory. With this background, it is now possible to historically understand why Mitchell refused to accept the higher values of H^+^/O and H^+^/ATP ratios than the ones he had postulated in his theory, even in the face of the most incontrovertible experimental evidence, as documented by Prebble [49]. These stoichiometries were central and fundamental to chemiosmosis, and acceptance of the higher numbers would have created a severe energy crisis for the chemiosmotic theory and sounded the death-knell for it. Workers in the field accepted the new, higher stoichiometries obtained repeatedly by experiment, but did not ask the chemiosmotic theory to account for these higher stoichiometries and failed to take cognisance that the theory contradicted the experimental facts, despite the herculean efforts of Slater [50], Lehninger [51], Williams [52], Green [53] and other stalwart workers, probably because no alternative theory of the scope of the chemiosmotic theory was available at that time. Hence the old theory and the new experimental facts have co-existed uneasily, but the old theory cannot explain the new facts; therefore it is absolutely necessary to go beyond chemiosmosis, if we have to solve the fundamental discrepancies that exist between theory and experiment.

Any mechanism should finally also be able to withstand the ultimate challenge of satisfying the overall, macroscopic energy balance of cellular metabolism (keeping the constraints imposed by the oxidative phosphorylation process intact). Thus, we should investigate whether the overall energy balance for the complete oxidation of glucose and the cytoplasmic ATP yield in the cell is satisfied from the sides of energy supply and energy production [54]. For a basis of 1 mole of glucose, the energy available from the supply side for ATP synthesis is 672 kcal/(mol glucose). On the user side 38 ATP molecules are produced per mole of glucose. The efficiency of the oxidative phosphorylation machinery on the redox side, η_redox_, is the product of the mechanistic P/O ratio and the ratio of the affinity of phosphorylation and the affinity of oxidation (A_P_/A_O_), in accordance with the principles of nonequilibrium thermodynamics when the degree of coupling, q → 1 [20, 55, 56]. Taking the consensus experimental values of the mechanistic P/O ratio as 3.0 [2, 46, 47], as discussed in this section, and the experimentally obtained operating (A_P_/A_O_) affinity ratio in mitochondria in state 3 as – 0.26 [20, 57], we obtain the efficiency on the redox side, η_redox_ as 0.78. This value can also be derived theoretically from the torsional mechanism. The value of the affinity of oxidation, A_O_ for the respiratory chain in mitochondria is 2280 meV per two electrons, or 220 kJ/mol. The value of the affinity of phosphorylation, A_P_, according to the torsional mechanism is the 54 kJ/mol of stored energy in the entire process plus losses, especially due to untwisting of an α-helix in the inlet half-access channel of F_O_ upon its protonation (see Section 5.4 also), and the entry of the untwisted helix carrying the bound proton into the membrane, which was a required conformational change for rotation of the c-rotor [1, 12]. This is estimated to be approximately one-ninth of the energy donated by ion translocation in the inlet half-access channel for a c-ring with ten c-subunits, and since there is no corresponding loss in the exit half-access channel, it is approximately 1/18 of 54 kJ/mol or ∼3 kJ/mol. Hence the value of A_P_ is estimated from the torsional mechanism to measure 54 + 3 = 57 kJ/mol. Therefore, the estimated value of η_redox_ from the torsional mechanism is 3.0 × (57/220) = 0.777 (77.7%).

Now we are in a position to perform the overall energy balance of cellular bioenergetics. For a basis of 1 mol glucose, we have an energy production of 57 kJ/mol per ATP × 38 ATP per mol glucose = 2166 kJ/mol glucose. On the supply side, the energy available is 672 kcal × 4.18 kJ/kcal × 0.777 = 2182.5 kJ/mol glucose, which is exactly adequate, because making the next integral number of ATP molecules (39) would have required 2223 kJ/mol, which is more than the energy available from the supply side. Thus the overall balance of cellular bioenergetics is perfectly satisfied. The torsional mechanism can be employed to go further in that it can offer us an excellent estimate of the efficiency on the ATP side, η_ATP_. Since the value of A_P_ of 57 kJ/mol includes the energy required to torsionally strain a bond so that MgADP can bind, the energy required to bind P_i_, the energy required to make the ATP plus the small irreversible loss in the cycle, the efficiency of ATP synthesis is 36/57 or 0.632 (63.2%). This value of η_ATP_ is also in agreement with the experimentally measured efficiency of light-driven production of ATP catalyzed by F_1_F_O_-ATP synthase in a liposome-based artificial photosynthetic membrane [58]. Thus the overall efficiency of oxidative phosphorylation in mitochondria (from donation of energy by the redox processes to the energy finally stored in ATP), η_overall_ is the product of η_redox_ and η_ATP_, or 0.777 × 0.632 = 0.491, *i.e.* 49.1%. All the various aspects discussed in this and previous sections of the paper serve to give us complete confidence in the correctness of the mechanistic, thermodynamic and kinetic proposals within the framework of torsional mechanism of energy transduction and ATP synthesis/hydrolysis and the unified theory.

## 5. Beyond the Chemiosmotic Theory: Details of the Molecular Mechanism of Energy Transduction in the Membrane-bound F_O_ Portion of F_1_F_O_-ATP Synthase

### 5.1. Several Lines of Biochemical Evidence that Cannot be Accounted for by the Chemiosmotic Theory but Are Logically Explained by the Torsional Mechanism of Energy Transduction and ATP Synthesis

Recently, we have shown that several lines of biochemical evidence, many from our own laboratory, do not support the chemiosmotic theory, but are readily explained by the torsional mechanism of energy transduction and ATP synthesis. These include: (i) the acid concentration dependence of the rate of ATP synthesis [11], (ii) the isolation and characterization of several uncoupler-resistant mutants of oxidative phosphorylation, (iii) the increase in oxidative phosphorylation uncoupling efficacies with increase in lipid solubility of the uncoupler, other things remaining the same, and (iv) experimental data on the inhibition of ATP synthesis by known specific anion channel blockers such as the triorganotin compound, tributyltin chloride (TBTCl), and the stilbene compound 4,4′-diisothiocyanostilbene-2,2′-disulfonate (DIDS) [10]. The experiments on inhibition of ATP synthesis at nanomolar concentrations of the potent, specific anion channel blocker, DIDS were repeated with improved methods (Section 8) and similar results were obtained (Nath and Agarwal, in preparation). It is very difficult to explain why specific anion channel blockers inhibit the rate of ATP synthesis using theories such as chemiosmosis [6] that energetically link ATP synthesis to the translocation of a single agent only, *i.e.* protons. However, if ∼50% of the energy to synthesize ATP comes from anion translocation (by a small inorganic anion such as chloride or by an organic anion such as succinate which is singly charged under the experimental conditions in our plant system), and the other ∼50% is donated by proton movement through specific half-access channels in the F_O_ portion of ATP synthase, and both Cl^−^ (or succinate) and H^+^ move in a strongly coupled way (with 1:1 stoichiometry being the favored and most likely case) through access pathways that form a rigid intramembrane link, a structural union, as it were, in the membrane for the addition and joint utilization of energy and for regulation of transport and metabolism, as predicted by the torsional mechanism [1, 2, 10–13, 15], then such an inhibition in the concentration range of inhibitor that does not saturate the membrane binding sites is in fact the expected outcome. Thus, ATP synthesis becomes a multicomponent reaction involving anions and cations, and the F_O_ portion of ATP synthase is then an *A^−^–C^+^* *symsequencecotransporter*. A difference between the molecular mechanisms in mitochondria, chloroplasts, and bacteria is the nature of the anion (chloride, succinate) and cation (proton, sodium ion) to which the energy-transducing membrane is permeable.

**Figure 4.**
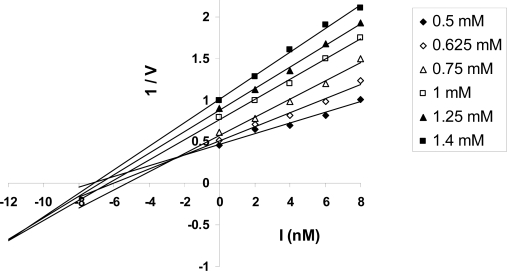
Dixon Plot of inverse of the rate of ATP synthesis measured in μmol ATP (mg chlorophyll)^−1^ min^−1^ (1/V) against anion channel inhibitor DIDS concentration (I) measured in nM for ATP synthesis by spinach thylakoids. Inhibiton was studied with HCl and DIDS concentrations ranging from 0.5 mM to 1.4 mM and 0 nM to 8 nM respectively. The HCl concentrations employed were: 0.5 mM (♦); 0.625 mM (⋄); 0.75 mM (▵); 1.0 mM (□); 1.25 mM (▴); 1.4 mM (▪).

For ATP synthesis by mammalian mitochondria, a singly-charged dicarboxylic acid anion such as succinate serves as the permeant anion *in vitro* and *in vivo* (distinct from its well-known role as a respiratory substrate in mitochondria). In chloroplasts, both small inorganic anions such as chloride [10] and organic acid anions such as succinate [11] support ATP synthesis at physiologically high rates *in vitro*. However, the narrow range of HCl concentrations in which measurable rates of ATP synthesis are found *in vitro* (Figure 4), the fact that chloride is the principal anion in plant chloroplasts present at high concentrations of ∼150 mM, necessitating an ultra-high intra-thylakoid concentration > ∼150 mM to generate a gradient that can support outward anion translocation and ATP synthesis in chloroplasts that have not been observed experimentally, the strong acid nature of HCl that may interfere with stability of ionic conditions in the chloroplast, the broader range of succinate concentrations that supports ATP synthesis (∼1 to ∼10 mM) at high rates [11], the clean exponential nature of the data [11], the weak acid nature of succinic acid, and finally the known experimental fact that chloride is essential only for those photosynthesis steps in which oxygen evolution occurs but is not necessary for cyclic photophosphorylation in bacteria and isolated chloroplasts involving photosystem I and ATP synthase (which we believe is operative under our acid-base/light-dark experimental conditions) all suggest that chloride is not the permeant anion *in vivo*. Since organic acids are the major products of photosynthetic activity, weak acid organic anion substances are the ubiquitous type of carbon compounds expected to be present at moderately appreciable concentrations (∼millimolar) within the chloroplasts *in vivo*, and hence the type of mechanism involving such organic acid anions and protons described above can be readily expected to occur *in vivo*. If this is so, then we also have a unity in the nature of the permeant anion that is translocated through the a-subunit access channel in F_O_ and supports ATP synthesis. It should however be noted that whether the specific permeant cation is H^+^ or Na^+^ and the permeant anion is Cl^−^ or a low molecular weight weak acid anion does not directly impact the torsional mechanism.

Inhibition studies [10] have helped to experimentally establish a role for anions in ATP synthesis. They have revealed the requirement of permeant anions (in addition to protons), and have indicated an energy provision role of the anion in bioenergetic coupling of ATP synthesis by translocation through the membrane-bound F_O_ portion and binding to the protein-in-the-membrane (*i.e.* trapping of chloride in its binding pocket in a lipophilic region of the membrane). The properties of these lipophilic regions have been considered and detailed molecular mechanisms developed within F_O_ [1, 2, 10–13, 15, 19]; such detailed explanations of the role of membrane elements have led to a deeper understanding and offer a more realistic and complete picture of biological energy transduction than a theory such as chemiosmosis [6] that strictly permits only “energized bulk aqueous phases” and considers the bilayer as “mere insulation”.

### 5.2. Establishment of the Type of Inhibition Found with Potent, Specific Anion Channel Blockers Such as DIDS and its Logical Explanation

It now becomes important to determine, classify and explain the type of inhibition found with the inhibitor (DIDS) at various HCl concentrations within the framework of the torsional mechanism. Inhibitor concentrations in the nanomolar range between 1 nM and 8 nM and HCl concentrations between 0.5 mM to 1.4 mM at which we could obtain measurable rates of ATP synthesis were chosen. Inhibitor and HCl at concentrations mentioned above were used to define the type of inhibition, and the result was inferred from a plot made between the inverse of the rates at a particular HCl concentration and different inhibitor concentrations. The results are shown in Figure 4.

Based on the cumulative observations depicted in Figure 4, the results were inferred as showing inhibition of a mixed type, which we suggest to be predominantly competitive with respect to chloride and predominantly uncompetitive with respect to chloride and proton, depending on the HCl concentration. Such a mixed type of inhibition has generally been encountered in multi-substrate reactions. The binary enzyme-inhibitor complex or ternary enzyme-substrate-inhibitor complex thus formed does not lead to any rates or results in decreased rates of enzyme activity. It is a virtue of competitive inhibition that once the inhibitor has bound to its site on the enzyme to form the enzyme-inhibitor complex, the ion/substrate for which the inhibitor is competitive has to remain in an unbound state irrespective of whether they share the same binding site or have different binding sites. On the other hand, a prerequisite condition for uncompetitive inhibition is that bound inhibitor cannot unbind and leave as long as the ion/substrate for which the inhibitor is uncompetitive remains bound to its site on the enzyme.

In our system, the inhibition can be competitive with respect to the primary ion, *i.e.* the one that binds/unbinds before the binding/unbinding of the second/secondary ion takes place, while it can be uncompetitive with respect to the primary ion, or with respect to both ions, *i.e.* both chloride and proton, which remain bound to their respective sites (e.g. if unbinding of the primary ion is required before the secondary ion can unbind, as in an ordered and sequential mechanism). Any mechanistic explanation has to be also consistent with the known role of DIDS as a potent anion channel blocker. In our mode of study, the DIDS inhibitor was added to the external aqueous medium on the stromal side of the membranes at the time of phosphorylation *i.e.* in the base stage. Hence we logically expect the DIDS to bind to its site on the membrane surface on the stromal side and block ion exit from the access channel/pathway and not interfere with ion entry from the intra-thylakoid space (lumen side). Moreover, since the inhibitor used in our experiments is a potent anion channel inhibitor, we infer that DIDS inhibition is competitive with respect to the chloride ion, and in fact all our results could be successfully explained by considering the ability of the chloride ion (i) to remain bound to its site or (ii) to unbind from its site but not exit from the access channel due to the presence of bound inhibitor at its own site. The inhibition can be classified as uncompetitive or competitive for the cases (i) and (ii) respectively. Thus, after both chloride and proton have bound to their binding sites on the a- and c-subunits respectively at the a–c interface of the F_O_ portion of the F_1_F_O_-ATP synthase, the inhibition would be competitive with respect to chloride if the Cl^−^ unbinds from its site but cannot come out of the a-subunit anion exit access channel due to the block caused by binding of DIDS to its site at the surface of the a-subunit anion access channel. The inhibition would be uncompetitive with respect to chloride if the chloride is unable to unbind from its site at the HCl concentrations employed, and uncompetitive with respect to both Cl^−^ and H^+^ if the chloride ion cannot unbind from its binding site, and if the two ions translocate in a coupled way and the H^+^ can only unbind following unbinding of the Cl^−^, in which case both Cl^−^ and H^+^ will remain bound to their binding sites on the a- and c-subunits respectively. One would expect the tendency of the anion to remain bound to its site in the a-subunit access channel to be readily subject to modulation by the stromal side concentrations of the substrate for the F_O_ portion of the F_1_F_O_ (e.g. HCl or succinic acid).

The above analysis has summarized the inhibition of ATP synthesis in the presence of anion channel blocker as competitive or uncompetitive depending on the tendency of the primary ion bound to its site to unbind and leave or to remain bound to its site. This tendency itself will be a strong function of the concentration gradient of the substrate for which measurable rates can be obtained in thylakoid membranes. At lower HCl concentrations (0.5 mM to 0.75 mM), mixed inhibition that is predominantly competitive has been found (Figure 4), and the second, minor component of this mixed inhibition has been characterized in the above discussion as uncompetitive in nature. At higher concentrations of HCl (1 mM to ∼1.5 mM), the inhibition was also interpreted to be mixed inhibition but predominantly uncompetitive in nature (Figure 4) while the minor component of this inhibition was characterized as competitive.

In our experiment, we have a large population of thylakoids (approximately 10^12^ per mg chlorophyll) and further there would be a large number of sites in each thylakoid. Taking only F_O_ sites that contain bound inhibitor, there will be heterogeneity of the ionic microenvironment around such sites and in particular, we shall only have a statistical distribution of chloride ions. Each of the ∼10^12^ thylakoids per mg chlorophyll in the isotonic medium cannot be expected to be perfectly swollen if the ion permeation process in both acid and base stages is intrinsically statistical in nature. This will lead to heterogeneity in the number of chloride ions around each chloride binding site. Finally, the microdistribution of ions among the F_O_ sites in each thylakoid will not be perfectly uniform leading to further spatial heterogeneity in ionic distribution. Hence, even in experiments tailored to unbind chloride from its binding site in each F_O_, there will always be a small population in which chloride does not unbind due to the above mentioned heterogeneity. In other words, only the average bulk concentration (but not the local number of Cl^−^ ions around each binding site) can be controlled by the experimentalist.

Hence, at low HCl (∼0.5 mM), Cl^−^ will unbind from a majority of the sites and lead to competitive inhibition in the presence of inhibitor; however, due to fluctuations in the stromal chloride concentration around the exit access channel, Cl^−^ will be unable to unbind and this will cause uncompetitive inhibition in a minority of sites containing bound DIDS.

At higher average chloride stromal concentration the situation is the reverse. Chloride remains bound in the majority of the sites because the driving force for unbinding of chloride is low. Hence, we have the presence of an ESI bound form and a predominantly uncompetitive inhibition. In a minority of sites, due to special heterogeneity for the reasons discussed above, the Cl^−^ ion can unbind from its binding site but cannot exit from the access channel due to the presence of bound inhibitor, and therefore we have a competitive inhibition with respect to chloride in such sites. It should however be emphasized that despite the presence of heterogeneity, the data (Figure 4) show that predominantly we do obtain the type of inhibition that we expect, implying the distribution of Cl^−^/H^+^ is not too nonuniform. Due to the reasons discussed above, it would be difficult to obtain pure competitive/uncompetitive behavior even in a single molecule experiment, *i.e.* to reproducibly simulate an all-or-none situation when chloride will unbind or remain bound to its site each and every time.

### 5.3. Explanation of Rate Enhancement of ATP Synthesis at Very High Inhibitor Concentrations

At very high concentrations, [I], of tributyltin chloride (TBTCl) ([I] ≥ 100 nM) and 4,4′-diisothiocyanostilbene-2,2′-disulfonate (DIDS) (100 nM ≤ [I] ≤ 2 μM), a rate enhancement of ATP synthesis (∼8-fold with TBTCl and ∼2-fold with DIDS) was reproducibly observed [10]. An attempt was made to explain the observation of rate enhancement but this proved to be a difficult exercise in the absence of a proposal of a different mechanism or of a special phenomenon operating at these artificially high TBTCl or DIDS concentrations [10]. Here we propose that after unbinding from their respective binding sites on the a- and c-subunit in F_O_, because of lipid solubility at the high concentrations of TBTCl or DIDS (≥100 nM), the Cl^−^ and the H^+^ combine to form *neutral HCl* in the microenvironment of the exit access half-channel. The neutral HCl can relieve the block caused by bound [I], for example by diffusing out by *molecular diffusion*. We propose that such molecular diffusion occurs at enhanced rates compared to ion transport as individual H^+^ and Cl^−^ charges and thereby leads to more cycles of ATP synthesis per unit time. Moreover, neutralization by formation of HCl is electrostatically akin to removing H^+^ and Cl^−^ to infinity. Hence no inhibition is observed at these conditions, and since the rate of transport of molecular HCl is faster than that of H^+^ and Cl^−^ individually, a rate enhancement of ATP synthesis occurs, as observed [10]. It should also be understood that energy transduction is not hampered if H^+^ and Cl^−^ recombine *after* unbinding from their respective binding sites at the a–c lipid-water interface.

### 5.4. Precise Explanation of Uncoupling Action by Weak Acid Anion Uncouplers of Oxidative Phosphorylation

In the light of the discussion in Sections 5.1 – 5.3, the earlier explanation given for uncoupling action by weak acid anion uncouplers (U^−^) of oxidative phosphorylation [see Figure 3 of Ref. 11 and discussion below that Figure] can now be fine-tuned and made more precise. Since recombination of U^−^ and H^+^ after their unbinding does not dissipate ∼50% of the energy (Section 5.3), an uncoupler anion, U^−^ is one that combines with H^+^ due to the lipid solubility of the uncoupler and forms neutral UH in the microenvironment at or near the binding sites upon entry of U^−^ and H^+^ sequentially through their own access channels located in the a-subunit and c-subunit respectively at the a–c interface. The formation of neutral UH precludes the conformational change in the α-helix containing the H^+^ binding site (the Asp-61 residue in E. coli ATP synthase) from occurring. Therefore, entry of that helix into the membrane [1, 12] is prevented from taking place. a–c electrostatic interactions cannot take place until this helix buries its negatively charged Asp-61 and H^+^ charges inside the membrane, and without the disequilibration and the electrostatic interactions [1, 12, 15, 19], rotation of the c-rotor cannot occur. The neutral UH diffuses out, reaches the bulk aqueous phase, dissociates into U^−^ and H^+^, and the U^−^ and H^+^ are pumped against their concentration gradients by the electron transport chain in mitochondria, as explained earlier [11].

### 5.5. The Way Forward

Keeping the above discussion and the results of inhibition patterns of Figure 4 in mind, it was not possible to explain the role of anions for ATP synthesis identified in this work using previous models, such as the chemiosmotic theory. In a sense, this is clearly understandable, because chemiosmotic dogma and other models of energy coupling only consider the proton as the coupling ion, and do not invoke any role for other ions in energy coupling. Recent debate [29, 59, 60] has pointed out other long-lasting difficulties, inconsistencies, and ambiguities in the earlier theories. However, no way forward has been suggested [59, 60]. A mechanism that could explain all our experimental findings is the torsional mechanism of energy transduction and ATP synthesis [1, 2, 10–29]. This mechanism specifies the detailed events taking place in both the membrane-bound F_O_ and the extra-membrane F_1_ portions of the enzyme and their temporal order during ATP synthesis. A unique tenet of this mechanism is the significance and energetic role accorded to membrane-permeable anion translocation (apart from proton/cation translocation) in providing the total energy needed for the synthesis of ATP. The binding and unbinding of sequential anion and proton translocations in their respective half-access channels which are physically located close to each other, the coupled nature of such transport, and the *local and direct* energy transduction taking place has been described in consummate detail in the mechanism [1, 2, 10–13, 15, 19]. The Δψ envisaged in the new theory is localized inside the half-access channels and is created by each elementary act of anion and proton binding to and unbinding from its binding site on the a- and c-subunit respectively at the a–c interface [10–13, 15]. This *localized Δψ* (∼35–45 mV due to each elementary act of anion/proton binding or unbinding to/from their respective half-access channel) has no relationship with the large *delocalized Δϕ* (∼120–180 mV in mitochondria) across bulk aqueous phases presumed to exist in chemiosmosis. (Since both Δψ and Δϕ are symbols for electrical potential differences, but have entirely different meanings in this context, it is suggested that to avoid confusion, the symbol Δψ be used to represent the *local* electrical potentials of the torsional mechanism of energy transduction and ATP synthesis which are created and destroyed in aqueous access channels, while the symbol Δϕ continue to be employed to refer to the *delocalized* potential differences presumed to exist across bulk aqueous phases by the chemiosmotic theory). In the new paradigm, violation of electrical neutrality of bulk aqueous phases to the extremely and unrealistically large extent postulated in chemiosmosis by uncompensated, electrogenic proton translocation is avoided [10, 11]. Numerous difficulties with the chemiosmotic theory, and how they are readily overcome by the torsional mechanism, have been discussed earlier in considerable detail [1, 2, 10–13]. The major differences between the torsional mechanism and the chemiosmotic theory have been summarized in tabular form on pp. 152–153 in Ref. [2]. In the present paper, the explanations for the observed patterns of inhibition of ATP synthesis by potent, specific anion channel blockers (Figure 4) have been improved and ideally they serve to replace the previous explanation given in the second paragraph on page 2221 of Ref. [10]. Revision of the previous theories along the lines described in detail in the torsional mechanism and in the unified theory in this section provides a way out of the present impasse, and resolves the problems by its new molecular systems biology ideas and approaches once and for all [10], and allows us to go beyond the chemiosmotic theory, which is now outdated, having first come into existence ∼50 years ago, and can no longer provide a valid theoretical basis for further experimental advances or for the progress of further theoretical research in the field in the future.

## 6. Applications of the Unified Theory to Other (Processive) Molecular Motors Crucial to Cell Life

The cytoplasm of cells is crowded with cargoes that are being moved processively along tracks of microtubules or actin by various molecular motors of the kinesin, myosin and dynein family. Efficient translocation of vesicles and membranous organelles such as mitochondria and endoplastic reticulum over micrometer distances is an activity crucial to cell life. The best-characterized unconventional myosin motor stepping processively on actin filaments is myosin V, which is involved in several types of intracellular transport. Kinesin constitutes a very large family of motor proteins that transports cargo processively on microtubules at speeds as high as ∼1 μm/s, and there are as many as ∼50 different kinesins in humans [61]. The best-characterized so-called conventional kinesin is abundant in nerve cells. It is double-headed and has an N-terminal motor domain, a coiled coil stalk, and a C-terminal cargo-binding domain, and transports cargo processively towards the microtubule plus end, *i.e.*, toward the cell membrane. A central issue in the field of molecular motors has been to understand how the chemical and mechanical steps are coupled and how processive motion occurs using the energy of ATP hydrolysis in such conventional kinesin and myosin V motors. Various models have been developed and different mechanisms proposed to explain processive motion by these molecular motors.

Due to space limitations, it is not possible to study the scores of each and every variant of kinesin, ncd and myosin V models for processive motility and compare and contrast them. However, the various alternative mechanisms can be *classified*, their overall features understood, and the unified theory applied to the mechanism or model of choice. It is well known that kinesin, ncd and myosin V heads are structurally equivalent. Hand-over-hand models of processive movement postulate equivalence of the two heads, *i.e.* not that the heads are structurally equivalent, which is well-known and understood as stated above, but that the heads are *functionally equivalent*, *i.e.*, the same biochemical (especially ATPase) events occur in both heads except that these events alternate and lag in time. A subset of these models, called symmetric hand-over-hand models [62, 63] also include the key proposal of *step equivalence*, *i.e.* that all steps are generated in the same way. Thus in symmetric hand-over-hand models, the rotation is 180° in a clockwise or counterclockwise sense each time, and the stepping head passes the head attached to the track from the same side (left or right) each time. Thus, symmetric hand-over-hand models postulate head equivalence and step equivalence [62, 63]. However, experimental data from the Gelles group contradicted the above because the postulated 180° swiveling or rotation was not observed with each step [64]. To explain these observations, head equivalence was abandoned and an inchworm model was proposed [64] in which the heads were *functionally inequivalent* in that only one head was an active ATPase, and one head always led the other, but the mechanism retained step equivalence. Thus the inchworm model is head inequivalent but has step equivalence. Based on single molecule experiments, Block and colleagues postulated an asymmetric hand-over-hand model of kinesin movement [65] in which the two heads are functionally equivalent and both hydrolyze ATP alternately, but the *steps are non-equivalent* in that the stepping head passes the attached head on alternate sides and the stalk makes compensatory movements to suppress the 180° rotations. Hence the asymmetric hand-over-hand model has head equivalence but does not have step equivalence. However, the head equivalence postulated by the asymmetric hand-over-hand model is challenged by the observation that heterodimers with ATPase mutation in only *one* kinesin head move processively [66]; in other words, processive motion is possible without alternating catalysis. Further, there exist difficulties for the asymmetric hand-over-hand model in explaining the exact cause of limping by homodimeric kinesin [65] in which no structural asymmetry is apparent. Based on molecular systems biology approaches, Nath has proposed a rotation-twist energy storage mechanism for processive molecular motors such as kinesin, ncd and myosin V [11], which was subsequently developed in further detail [10]. In this mechanism, one head of the motor binds weakly to the α-tubulin subunit on the microtubule or to actin while the other head binds strongly to the β-tubulin subunit on the microtubule or to actin. To take the example of kinesin, only the head bound to the β-tubulin subunit on the microtubule is an active ATPase because the interactions of the α-tubulin with the bound kinesin head are not strong enough to release the MgADP bound in that head; hence no ATP hydrolysis occurs in the head bound to the α-tubulin subunit on the microtubule. Further, the two steps occur due to different causes; the first one occurs due to ATP binding and its subsequent hydrolysis (called “the chemical stroke” [10, 11]), while the cause of the second step is the torsional strain in the V-shaped molecule up to hinge-1 due to the ATP hydrolysis Coulombic energy released upon P_i_ release, though the step itself takes place after the trigger of ADP release from the head and the release of the torsional strain (called “the physical stroke” [10, 11]). In the physical and chemical strokes, the rear head passes the bound front head from different sides and the strokes have different trajectories; moreover, since the strokes originate from different causes, they differ in their kinetics, and thus the rotation-twist energy storage mechanism for processive molecular motors has *head inequivalence and step inequivalence*. The mechanism has no difficulty in explaining the observations in Ref. 66 because in the mechanism, only one head hydrolyzes an ATP molecule to move the double-headed molecule processively by ∼8 nm, and further, the progressive shortening of the coiled coil in the truncated constructs [65] hamper the storage of elastic strain in the molecule which adversely affects the performance of the physical stroke and therefore increases the propensity of the molecule to limp. The mechanism is simpler and more elegant and is ideally expected to operate *in vivo*. The mechanism can readily be adapted to the case *in vitro* when both heads bind to β-tubulin subunits on the microtubule, the only difference being that ATP binding will now be required to unbind the second head also (*i.e.* the head that had bound earlier to the α-tubulin subunit on the microtubule), as discussed later, in which case the mechanism can be said to have head equivalence but no step equivalence. It should be noted that there is considerable experimental evidence for specific interaction of kinesin and ncd motor heads with both α- and β-tubulin [67–70]. Moreover, the weaker affinity of the specific binding site on α-tubulin may prevent its identification, or it is likely that both the heads may preferably bind to the higher-affinity specific sites on β-tubulin *in vitro*, and another positive point of the new mechanism is that it assigns a role to the second weaker but specific binding site identified by biochemical labeling and electron microscopy techniques [67–70] in motor function. The function of these weaker, specific motor-binding sites on the microtubule that have been revealed by experiment had hitherto been unknown. Finally, it should also be noted that the inchworm model was also defective because it did not solve the difficult problem of how the motor moved both heads by hydrolyzing a single ATP molecule. This is not a defect in the new mechanism.

Recent fluorescence labeling and single molecule studies from Selvin’s group have clearly invalidated the inchworm model of processive motion and shown that kinesin moves in a hand-overhand fashion [71]. Other models have also been proposed in which the two heads are 4 nm apart [72, 73], and Hackney’s classical experimental data also mean that there are two motor head binding sites with *different* affinities on the microtubule [74, 75]. These models are unable to explain the 16 nm steps seen in single molecule studies, which imply that the two heads of kinesin are 8 nm apart [71]. The limping seen in homodimeric kinesin has proved very difficult to explain, and Selvin and colleagues have recently stated that kinesin could indeed walk symmetrically and that the observed limping was due to the experimental conditions [76]. Unfortunately, their explanation contradicts the experimental data of the Gelles group [64]. Impressive evidence that kinesin moves by an asymmetric hand-over-hand model has also been produced recently [65, 77], and, in particular, Block and colleagues favor a gated front head model [77].

However, in addition to the question of limping by homodimeric kinesin constructs which has not been satisfactorily answered to date by the asymmetric hand-over-hand model of motility, there is another even more fundamental and central question that has not been asked till now, but needs to be asked now and sorely requires an unequivocal answer. This question is the following: Does kinesin move forward processively in steps of 16 nm head-to-head (*i.e.* 8-nm steps for movement of the center of mass of the double-headed molecule) *against a load of ∼7 pN*, the *maximum* force against which kinesin molecules have been consistently recorded to step forward in various expertly-performed state-of-the-art single molecule experiments [72, 78–80], and not ∼half the load of ∼7 pN, *i.e.* ∼3.5–4 pN, where it is certainly known to move forward processively with 8-nm (center of mass) steps [65]? Answering this question is crucial because in the optical trap experiments, for constant energy input from ATP, it is readily possible to satisfy the energy balance by reducing the force by a factor of ∼2 and thereby obtain an increase in the step size by a factor of ∼2. In a way, this question has already been answered by single molecule experiments on kinesin at an *intermediate* force range of 4.5–5.3 pN, where already, kinesin starts to step backwards [77]. Hence the all-important question arises: Why does kinesin step backwards at intermediate loads of 4–5 pN, well *below* the experimentally measured stall force of ∼7 pN? In fact, it should have stepped *forward* processively even at a ∼7 pN rearward load, in accordance with the single molecule experiments of several groups, including Block’s own group [72, 78–80]. Unless the step size (8 nm based on center of mass movement) in these *in vitro* experiments is *larger* than the actual step size *in vivo*, and therefore the energy of ATP is *insufficient* to supply the energy for processive forward stepping against the intermediate rearward load of *even* 4–5 pN (let alone ∼7 pN), and therefore the rearward load wins and pulls the kinesin molecule backwards, it is not possible to explain the observation of backward stepping at these intermediate loads, according to our analysis. In the absence of a demonstration of processive forward stepping with 16 nm head-to-head steps at the *maximum* load against which kinesin molecules are known to move forward processively [72, 78–80], the proposal of hand-over-hand models that the molecule always advances by 16 nm head-to-head steps (8 nm movement of the center of mass) is significantly weakened. I predict that this question will prove to be the Achilles heel for the asymmetric hand-overhand model. Ideally, what is needed is a molecular mechanism for forward processive motility that works up to the maximum force (∼7 pN for kinesin) and not a mechanism that works only up to ∼half the maximum force, and when the load is increased slightly beyond this value, the motor begins to move in the backward direction. The rotation-twist (RT) energy storage mechanism for processive molecular motors offers the further advantage that it explains forward processive motion under *vertical loading* that we expect *in vivo* due to the presence of the cargo at the C-terminal end of kinesin. It should also be pointed out that the asymmetric hand-over-hand model is not the only model that includes alternation between two different configurations during processive stepping; the RT mechanism also shares this attribute [10, 11]. Hence we believe that the rotation-twist (RT) energy storage mechanism for processive molecular motors should also be included among candidate mechanisms in the field of motility along with symmetric and asymmetric hand-over-hand and inchworm models.

The RT mechanism is readily able to explain the experimental facts [62–80] and also possesses several new and attractive features. Thus the data on processive movement of kinesin against the maximal load of ∼7 pN [72, 78–80] is explained by the RT mechanism by postulating that in these experiments the two kinesin heads must have bound to α- and β-tubulin on the microtubule. In the experiments in which the two heads are bound to adjacent β-tubulin subunits on the microtubule, forward processive motion can readily occur at a load value up to approximately half the maximum load, with the larger step size of 16 nm (head-to-head), as observed [65]. Thus, forward processive motion is possible in both cases, but not against the high ∼7 pN load in the case when both kinesin motor heads bind to β-tubulin subunits on the microtubule. Thus, from the point of view of the RT mechanism, kinesin can move with 4 nm center of mass (= 8 nm head-to-head) steps or with 8 nm center of mass (= 16 nm head-to-head) steps. The two cases depend on whether one head binds to β-tubulin and the other to the adjacent α-tubulin subunit of the microtubule track (former case) or whether both heads bind to adjacent β-tubulin subunits of the microtubule track (latter case). Since a motor head is bound weakly to α-tubulin (compared to the binding of the counterpart motor head bound to β-tubulin), it does not require MgATP binding to help its release from the microtubule, as the stored strain energy in the molecule after the chemical stroke and release of P_i_ is sufficient by itself to release such a (rear) head bound to α-tubulin [10, 11]. This is also consistent with the longstanding observation of the presence of two motor head binding sites with different affinities on the microtubule [74, 75]. On the other hand, if both motor heads bind to β-tubulin, then the stored strain energy is not sufficient to release the rear head bound to β-tubulin and requires help from another energy source, which we propose to be MgATP binding to the rear head in the RT mechanism. Applying the unified theory to the RT mechanism, we can state with full confidence that the first (rear) head is moved (past the bound front head) from one side by a clockwise rotation viewed from the C-terminal cargo end of conventional kinesin due to the ∼9 kJ/mol MgATP binding energy (*i.e.* the surplus energy released over that required to unbind the head from the microtubule) plus the ∼9 kJ/mol energy released upon the process of hydrolysis of the MgATP (that had just bound in that head) that occurs when the head is free from the microtubule track (and before P_i_ release, which requires binding of the head to the next binding site on the track). This is the “chemical step” [10, 11]. In the “physical step” [10, 11], the Coulombic repulsion energy between MgADP and P_i_ released upon P_i_ release into the medium (∼18 kJ/mol according to the unified theory) causes an elastic (primarily torsional) strain that helps move the second head (the present rear head) past the bound front head from the other side (compared to that in the earlier “chemical step”) by a counterclockwise rotation viewed from the C-terminal cargo end either without requiring MgATP binding in the second (rear) head in the case when the head is bound to α-tubulin, or also requiring MgATP binding in the rear head to help release the head in the case when it is bound to β-tubulin. The limping of homodimeric kinesin [65] is readily explained by the RT mechanism because as the kinesin stalk is progressively shortened, the energy storage process in the molecule is progressively hampered and the torsional rigidity required to generate torque by the physical stroke about hinge-1 [10, 11] is progressively compromised.

In the RT mechanism, MgATP binding to the *rear* head releases binding energy which unbinds the *rear* head from the microtubule track and gives it a clockwise torque (seen from the C-terminal cargo end), and it is perfectly reasonable that when the head starts to slow down or even pauses, as the sharp input of energy due to the release of MgATP binding energy in the head that initiated the chemical step gradually loses its effect, bond cleavage of MgATP occurs in the head and the release of the requisite quantum of hydrolysis energy (as detailed in the unified theory) continues to drive the head to its next binding site on the microtubule and helps it complete the chemical step, *i.e.* MgATP binding and MgATP hydrolysis acting in sequence cause the chemical stroke. This is a superior proposal to the central tenet of the gated front head model in which MgATP binding to the *front* head causes the *rear* head to step forward [77]. The RT energy storage mechanism also differs from the gated rear head model in which P_i_ release from the *rear* head (but not ATP binding to the rear head or the subsequent ATP hydrolysis that is postulated to occur in the rear head while it is still bound to the microtubule) causes the *rear* head (now containing bound ADP) to step forward past the bound front head. In both gated front head and gated rear head models within the hand-over-hand mechanisms, internal strain in the kinesin molecule is *only used to reduce the affinity* (of the leading head for ATP in the gated front head model, or of the rear head for the microtubule in the gated rear head model). It is only in the RT mechanism that elastic (twisting) strain energy is *stored* in the molecule and used to cause the forward stepping of the rear head past the bound front head upon release of the stored strain energy, *i.e.*, for performance of useful external work, which in our opinion is a great leap in our thinking and a progressive step forward for models of processive motility. In this sense, the RT energy storage mechanism and the symmetric or asymmetric hand-over-hand models belong to different classes of models of processive motility.

Finally the new molecular mechanism for processive motility is simpler, contains fewer biochemical steps, is aesthetically pleasing and elegant, and, especially significant in the context of processive motors, *faster* than previous models of processivity. The aspects discussed in this section are also applicable to unconventional myosins, and in fact, the mechanism has also been readily adapted to explain the processive motion of unconventional myosins such as myosin V on actin filaments [10, 11] and in the field of myosin motility, it may play an important progressive role. A key achievement of the RT energy storage mechanism in unconventional myosin motility is that it offers an increase of efficiency of intracellular transport by a *factor of two* over all previous mechanisms, a very large and significant improvement [11]. This is because, in all previous mechanisms, two ATP molecules are consumed for a forward movement of the center of mass of the double-headed myosin by 72 nm, while in the RT energy storage mechanism only a single ATP molecule is used to power the center of mass of the myosin forward by 72 nm. An identical rotation-twist energy storage molecular mechanism applies to the motility of the non-claret disjunctional (ncd) motor, with *one extra turn* in the ncd neck coiled coil (compared to kinesin) constituting the relaxed state of ncd, thus accounting for movement of the ncd motor towards the minus end of the microtubule. In conclusion, it can be stated that this section has clearly indicated a large number of useful applications of the unified theory to various processive molecular motors involved in intracellular motility, which is crucial to cell life.

## 7. Possible Applications of the Torsional Mechanism of ATP Synthesis and the Unified Theory in Apoptosis, Cell Death and Disease

As shown above, a comprehensive new theory of cellular ATP production has emerged through the proposal of Nath’s torsional mechanism of energy transduction and ATP synthesis using original systems biology/engineering approaches. In addition to providing the detailed molecular mechanism of ATP synthesis by F_1_F_O_-ATP synthase, the torsional mechanism has explicitly emphasized and quantitatively analyzed the key role of the *transport* steps through the adenine nucleotide translocase (ANT), the P_i_/OH- antiporter and F_O_ preceding ATP synthesis by F_1_, and has specified their exact sequential order as a law [1, 2, 11, 13]. The mechanism visualized the nature of these events as localized, coordinated and ordered in time and space by the (local) electrical potentials induced by these transport steps in their real molecular setting in the inner mitochondrial membrane. Recently, the isolation and characterization of the mitochondrial ATP synthasome as a 1:1:1 supercomplex of ANT, P_i_/OH^−^ and F_1_F_O_ located near one another in the membrane has been accomplished [48]. This finding of co-localization contradicts the classical view that regards these components as separate entities in the energy-transducing membrane and among all candidate mechanisms only the torsional mechanism is in accord with these important new data on membrane association of these entities to form the ATP synthasome supercomplex.

We now ask the question why apoptotic cell death by the intrinsic pathway is *initiated* in the mitochondria of cells [81–87]. This has generally been considered as a paradox, *i.e.* why organelles that are central to cell life are also involved in apoptosis and cell death. Either apoptosis is an ATP requiring process (but then this must be in the cytoplasm, e.g. in the apoptosome), or there is a need to block/inhibit and consequently shut down the energy transduction processes in the mitochondrion and this is a central and primary process in the intrinsic apoptotic pathway [81–84] or in the action of viral accessory proteins such as Vpr (Viral protein R) from Human Immunodeficiency Virus HIV-1 [85] or PB1-F2 from Influenza A Virus IAV [86, 87]. Otherwise there is no logical reason why the mitochondrion should be involved. Currently there is a substantial body of experimental evidence that implicates a direct and specific interaction of pro-apoptotic viral peptides such as Vpr from HIV-1 and PB1F2 from IAV with the ANT in mitochondria [81–87]. We propose here that *pro-apoptotic* agents achieve their effect by *inhibiting* mitochondrial ATP production via the ATP synthasome. The modes of inhibition can be further divided into classes of apoptotic agents/viral proteins that inhibit ANT, P_i_/OH^−^, F_1_F_O_ or form pores in the membrane. Since according to the torsional mechanism, the availability of ADP is the *rate-limiting step* of ATP synthesis, it is further proposed that inhibition via ANT, either by blocking exit of ATP from it or entry of ADP through it, *i.e.* by blocking nucleotide exchange through ANT is the *most economical way* of achieving the postulated reduction in mitochondrial ATP generation, and thereby reduction of cellular ATP levels, leading to an overall bioenergetic deficit in the entire cell and apoptosis through subsequent downstream events. It is also proposed here that intrinsically/*in vivo*, Vpr and PB1-F2 have an *anti-apoptotic* function and act by sequestering the amphipatic α-helix of BH3 regions of pro-apoptotic protein members of the Bcl-2 family such as Bax and/or Bak. However, in the absence of these pro-apoptotic members, Vpr and PB1-F2 lose this anti-apoptotic function, especially at the relatively higher concentrations in various experiments [85–87], and act as mimics of the pro-apoptotic members of the Bcl-2 family and themselves cause apoptosis. The oligomerization properties of these viral peptides may be vital for this function.

But the question arises why Vpr from HIV-1 and PB1-F2 from IAV should promote apoptosis in the *in vivo* situation? In a stressed condition, *i.e.* the pro-apoptotic state, the Bax/Bak proteins are activated through the absence or inability of the anti-apoptotic Bcl-2/Bcl-x_L_ family of proteins to maintain the pro-survival state of the cell. Therefore there is no natural anti-apoptotic agent available that can antagonize and stop the natural apoptotic response, which leads to cell death. However, the virus requires the host machinery to continue functioning, thereby allowing further viral production. As a consequence, we surmise that in the above context, the viral proteins function in an *anti-apoptotic* capacity by interaction and binding with Bax through neutralization of its BH3 domain, by analogy with the known interactions of the anti-apoptotic Bcl-2 family of proteins with the BH3 domains of Bax/Bak-like proteins. Thus, the Bcl-2 or its homolog Bcl-x_L_ family of proteins achieves its anti-apoptotic effect by maintaining Bax in an inactivated state and preventing Bax from exerting its apoptotic role.

What then could be the exact mechanism by which Bax induces apoptosis under conditions when it is freed from its direct physical interaction with Bcl-2/Bcl-x_L_, which was responsible for its inactivation, as discussed above? In our systems model of apoptosis, we propose a subtle and specific direct interaction of Bax with ANT, which is responsible for inhibiting the enzymatic function of ADP^3−^/ATP^4−^ exchange of the transporter, and is in agreement with a previous proposal [83]. In our view, this action of Bax on ANT is an essential primary event that is sufficient to cause and propagate the later events in apoptosis.

Why is it necessary for the natural apoptotic program or for various pro-apoptotic agents to inhibit ATP production and what consequences does it have for apoptosis by the intrinsic pathway? To the best of our knowledge, there is no experimental evidence in the literature that either the natural apoptotic program or pro-apoptotic agents *directly* target the electron transport or respiratory chain in mitochondria. In our view, the only natural way (*i.e.* without the experimentalist’s intervention) to *inhibit the redox side*, especially since electrons can readily tunnel through quantum mechanically, is to *inhibit the process to which it is coupled i.e.*, ATP synthesis and especially ion translocation (H^+^ and A^−^) (Section 5). If this flow of ions is inhibited then the redox activity (which would have actively transported these ions) is consequently inhibited too. Once the electron transport chain is itself inhibited, we can expect the water-soluble cytochrome c to accumulate in the intermembrane space. If the outer mitochondrial membrane is ruptured or if access channels in the outer membrane are open, then cytochrome c can be readily released along its concentration gradient from the intermembrane space to the cytoplasm, which then is the signal for initiating further downstream apoptotic events/cascades such as caspase activation and the various processes in the apoptosome.

Finally, on the basis of the torsional mechanism and the unified theory, *diverse* experimental data in the apoptosis/necrosis field can be naturally explained, including the role of oligomycin as a powerful inhibitor of apoptosis, the lack of any significant effect of azide on apoptosis, the effects of atractyloside, the local hyperpolarization/decrease of local depolarization in the vicinity of the the ATP synthasome induced by certain ester anions and other agents etc. If the above hypothesis of the involvement and role of ATP inhibition in apoptotic cell death via the intrinsic mitochondrial pathway is correct, then the occurrence of fundamental cell life as well as cell death processes in the same organelle is not “paradoxical” (as often stated), but is in fact a logical requirement for the desired function in both cases. Cell life and cell death can then be viewed as two sides of the same coin and studies of one side can synergize, impose further constraints, and truly help understand the other side, with manifold applications for both health and disease. Furthermore, failure of the apoptosis program will lead to irreversible disease, including cancer, via altered (e.g. glycolytic) pathways, and mechanisms for the resistance and lowered apoptotic potential of tumor cells, and reversal of the ATP synthase that is known to occur in such diseases and in necrosis can then also be further developed and conceptually understood based on the detailed ATP hydrolysis mechanisms that have already been postulated in the new framework of the unified theory and shown to be a microscopic reversal of the torsional mechanism of ATP synthesis. The cellular mitochondrial vs. glycolytic potentials may then provide a bioenergetic index for a logical assessment of carcinogenesis and the progression of carcinoma. In summary, a systems-level biological understanding of the torsional mechanism of energy transduction and ATP synthesis and the unified theory in cell life suggests a *bioenergetic* basis for mitochondrial apoptotic cell death and disease, and a large number of applications of our fundamental work in cell life are possible in cell death and disease also.

## 8. Experimental Section

### 8.1. Isolation of Thylakoid Membranes

Thylakoid membranes were isolated from spinach (*Spinacia oleracea*) leaves purchased from the local market by the method of Tripathy and Mohanty [88], as described earlier [11]. Briefly, spinach leaves were purchased and kept refrigerated in darkness to maintain chloroplast activity. Leaves were first deveined and a total of about 50 g of spinach leaves were washed with distilled water, kept at 4°C, soaked on filter paper and then homogenized in a Waring blender for 40 s together with the isolation buffer (Sorbitol 0.4 M, Tris 0.05 M, EDTA 1mM, MgCl_2_ 1 mM, adjusted to the pH 7.3) in the ratio of 1:8 w/v of spinach leaves to isolation buffer. The mixture obtained was filtered through 8 layers of cheesecloth and one layer of miracloth. The filtrate obtained was centrifuged at 1170 g for 7 min at 4°C. The supernatant obtained was discarded and the pellet which contained chloroplast, broken cell wall, starch grains was suspended in hypotonic TE buffer (Tricine 0.01 M and EDTA 1 mM, pH 7.5) in the ratio of 5:1 w/v of spinach leaves taken initially and kept in ice for 15 min. The suspension was again centrifuged at 4650 g for 5 min at 4°C. The supernatant was carefully discarded and the pellet containing thylakoid membrane along with cell debris and starch layer was dissolved in a minimal volume of hypertonic suspension buffer (Sorbitol 0.4 mM, Tris 0.05 M, EDTA 1 mM and 1 mM, pH 7.5) in 5:1 w/v of spinach leaves and finally stored at −80°C until used (for a MgCl_2_ maximum of 24 h). The whole procedure was carried out in the dark.

### 8.2. Estimation of Chlorophyll Content

The chlorophyll content was measured according to the method of Arnon [89]. The thylakoid membrane suspension (100 μL) was suspended in 80 % (v/v) acetone (20 mL) and filtered in the dark through Whatman no.1 filter paper. The optical density of the filtrate was determined at 652 nm against a blank of 80 % (v/v) acetone in a quartz cuvette and multiplied by a factor of 5.8 to give the chlorophyll concentration in mg/mL. The chlorophyll concentration was adjusted to 0.5 mg/mL with suspension buffer and was used for carrying out phosphorylation as described next.

### 8.3. Measurement of ATP Synthesis

The estimation of rate of ATP synthesis was carried out by phosphorylation of ADP by the technique of ‘acid-base’ transition [90]. The method was based on microcolorimetric estimation [91] of removal of phosphorus from the mixture, which has utilized for the formation of ATP from ADP. The classical, ‘acid – base’ transition scheme involves two steps: acidic stage and basic stage. In acid stage (AS) 0.5 mL of thylakoid membranes were incubated with 0.9 mL of acid (pH 4.0) for the desired time in order to accumulate the acid of interest. This was then transferred to 0.9 mL basic stage (BS) buffer (Tricine 100 μmoles, adenosine diphosphate 0.2 μmoles, KH_2_PO_4_ 2.0 μmoles, MgCl_2_ 5.0 μmoles, and NaOH 19.6 μmoles, pH 8.3) by 1:1 dilution, consequently allowing diffusion through the membrane from inside to out according to the concentration gradient of the species for catalytic synthesis of ATP via the ATP synthase complex. The reaction was terminated by the addition of 200 μL of 20 % (w/v) trichloroacetic acid (TCA). For the control, TCA was added in the base stage (prior to incubation with thylakoid membranes). The whole suspension (2.0 mL) was taken in two different tubes (1 mL each) and centrifuged for 10 min at 7270 g. Transfer of thylakoid membranes was carried out with 1 mL glass syringe attached with 20-gauge cannula, and the entire procedure was carried out in complete darkness. Inhibition studies were carried out with the inhibitor 4,4′-diisothiocyano-stilbene-2,2′-disulphonic acid (DIDS) added during the preparation of base stage buffer at different concentrations required.

### 8.4. Estimation and Calculation of Rates of ATP Synthesis

50 μl aliquot from each reaction mixture after centrifugation was mixed with 500 μl of colorimetric reagent (prepared freshly) as given earlier [91]. The OD at 660 nm was measured against sample blank for each reading. The decrease in phosphate concentration in test samples to that of control measured the ATP produced. The rate of ATP synthesis was calculated in μmoles of ATP produced (mg of chlorophyll)^−1^ min^−1^. Selected readings were also verified using ATP kit and luciferin-luciferase assay.

## 9. Prospects for Future Research

A reader who has persisted till this point will no doubt realize that this paper has dealt with some of the most fundamental processes in biology, namely ATP synthesis, ATP hydrolysis, muscle contraction, intracellular transport, and apoptosis and cell death and that each of these fields is extremely vast and that each will have its own agenda and directions for future research. Moreover, during the last decade, the immensity of each field and the ever-accelerating pace of new advances have seen the emergence of myriad specialized sub-fields (dedicated for instance to a particular molecular motor or to a particular disease) in an attempt to keep up with the latest developments and run a manageable research program. Each of these sub-fields has its own prospects and scope for further research and it would take a lot of space to enumerate them, let alone discuss them. Hence, in the interest of brevity, of what has already been a long paper, I shall desist from doing all of the above and restrict myself to exploring prospects for research in ATP synthesis/hydrolysis and oxidative phosphorylation in the immediate future. For structural biologists in the field, solution of a high-resolution structure of the complete F_1_F_O_ would present the next major challenge, which will also help in understanding mechanism of the complete synthase. However, biochemists and biophysicists who are not crystallographers need not lose heart, because the vast majority of studies in this field to date have been carried out in the hydrolysis mode, and a massive amount of experimental data has been generated; in contrast, information on the mechanism of ATP synthesis by F_1_F_O_ in the presence of ion gradients is extremely scarce. Hence, in the near future there is a great need to carry out biochemical and biophysical studies in the ATP *synthesis* mode in a bid to verify current hypotheses and to further understand crucial mechanistic issues and also give theoreticians something concrete to model in the ATP synthesis process. In turn, the torsional mechanism can catalyze this goal by serving as a guide for future experimentation. Despite several pioneering structural and single molecule studies on F_1_-ATPase, we still do not really know the answer to the fundamental question of how γ rotates, *i.e.* how force is produced by molecular interactions in the *hydrolysis* mode, and this aspect requires continued invesigation. Paradoxically, we can visualize force production and rotation of the c-rotor and the γsubunit in a better way in the *synthesis* mode, thanks to charge geometries and models founded on the basic principles of electrostatic theory [1]. However, these models need to be extended to include rotation in ∼15°/18° sub-steps resulting from the presence of two half-access channels and also to further take into account the energy storage properties of the enzyme during these sub-steps. Such analytical solutions or numerical simulations using the principles of engineering mechanics and dynamics is a worthwhile goal for the torsional mechanism, particularly because such quantification appears both attractive and feasible in the immediate future. There is also a pressing need for validation and further accurate determination of the stoichiometries of oxidative phosphorylation complexes I–V [92, 93]. Such information is essential for correct interpretation of kinetic and spectroscopic data and also for the development of structural models of supercomplex formation in mitochondria. On the redox side in oxidative phosphorylation, the central question of how electron-coupled proton translocation takes place has remained unanswered. Parallel to the new and exciting research efforts aimed at solving the molecular mechanism of ATP synthesis chronicled in this paper both comprehensively and in consummate detail, there have been a number of recent exciting experimental and theoretical attempts to elucidate the molecular mechanism of redox-linked proton translocation [94–97] but space does not permit us to delve into them here.

## 10. Conclusions

On the tenth anniversary of the proposal of the torsional mechanism of energy transduction and ATP synthesis, a unified theory of ATP synthesis and hydrolysis by the various biomolecular machines of the cell has been formulated. Complete details of the molecular mechanisms of the fundamental biological processes of ATP synthesis/hydrolysis and muscle contraction have been provided and the inconsistencies present in all previous theories have been removed. The new theory has been shown to be consistent with both the first and second laws of thermodynamics. The new mechanisms have been shown to be in agreement with the immense amount of experimental data in the literature and provide logical explanations and a theoretical basis for all the experimental observations. The novel framework has generated a large number of predictions that can be tested experimentally. Our theory is now ideally positioned to guide the design of new experiments, rationalize and interpret experimental data, and catalyze the progress of future theoretical research in this important interdisciplinary field.
